# Behavioral and Self-reported Data Collected From Smartphones for the Assessment of Depressive and Manic Symptoms in Patients With Bipolar Disorder: Prospective Observational Study

**DOI:** 10.2196/28647

**Published:** 2022-01-19

**Authors:** Monika Dominiak, Katarzyna Kaczmarek-Majer, Anna Z Antosik-Wójcińska, Karol R Opara, Anna Olwert, Weronika Radziszewska, Olgierd Hryniewicz, Łukasz Święcicki, Marcin Wojnar, Paweł Mierzejewski

**Affiliations:** 1 Department of Pharmacology and Physiology of the Nervous System Institute of Psychiatry and Neurology Warsaw Poland; 2 Section of Biological Psychiatry of the Polish Psychiatric Association Warsaw Poland; 3 Department of Stochastic Methods Systems Research Institute Polish Academy of Sciences Warsaw Poland; 4 Department of Psychiatry Medical University of Warsaw Warsaw Poland; 5 Department of Affective Disorders II Psychiatric Clinic Institute of Psychiatry and Neurology Warsaw Poland

**Keywords:** bipolar disorder, generalized linear model, mixed-effects regression, classification, manic episodes, depressive episodes, smartphone, behavioral markers, mHealth, remote monitoring

## Abstract

**Background:**

Smartphones allow for real-time monitoring of patients’ behavioral activities in a naturalistic setting. These data are suggested as markers for the mental state of patients with bipolar disorder (BD).

**Objective:**

We assessed the relations between data collected from smartphones and the clinically rated depressive and manic symptoms together with the corresponding affective states in patients with BD.

**Methods:**

BDmon, a dedicated mobile app, was developed and installed on patients’ smartphones to automatically collect the statistics about their phone calls and text messages as well as their self-assessments of sleep and mood. The final sample for the numerical analyses consisted of 51 eligible patients who participated in at least two psychiatric assessments and used the BDmon app (mean participation time, 208 [SD 132] days). In total, 196 psychiatric assessments were performed using the Hamilton Depression Rating Scale and the Young Mania Rating Scale. Generalized linear mixed-effects models were applied to quantify the strength of the relation between the daily statistics on the behavioral data collected automatically from smartphones and the affective symptoms and mood states in patients with BD.

**Results:**

Objective behavioral data collected from smartphones were found to be related with the BD states as follows: (1) depressed patients tended to make phone calls less frequently than euthymic patients (β=−.064, *P*=.01); (2) the number of incoming answered calls during depression was lower than that during euthymia (β=−.15, *P*=.01) and, concurrently, missed incoming calls were more frequent and increased as depressive symptoms intensified (β=4.431, *P*<.001; β=4.861, *P*<.001, respectively); (3) the fraction of outgoing calls was higher in manic states (β=2.73, *P*=.03); (4) the fraction of missed calls was higher in manic/mixed states as compared to that in the euthymic state (β=3.53, *P*=.01) and positively correlated to the severity of symptoms (*β*=2.991, *P*=.02); (5) the variability of the duration of the outgoing calls was higher in manic/mixed states (β=.0012, *P*=.045) and positively correlated to the severity of symptoms (β=.0017, *P*=.02); and (6) the number and length of the sent text messages was higher in manic/mixed states as compared to that in the euthymic state (β=.031, *P*=.01; β=.015, *P*=.01; respectively) and positively correlated to the severity of manic symptoms (β=.116, *P*<.001; β=.022, *P*<.001; respectively). We also observed that self-assessment of mood was lower in depressive (β=−1.452, *P*<.001) and higher in manic states (β=.509, *P*<.001).

**Conclusions:**

Smartphone-based behavioral parameters are valid markers for assessing the severity of affective symptoms and discriminating between mood states in patients with BD. This technology opens a way toward early detection of worsening of the mental state and thereby increases the patient’s chance of improving in the course of the illness.

## Introduction

Bipolar disorder (BD) is a chronic, recurrent, and highly morbid illness [1]. Its prevalence is estimated to be around 2%-3% [2]. In the course of the illness, there are fluctuations between different mood states, ranging from depression to hypomanic/manic episodes, as well as mixed states. Patients receiving mood-stabilizing drugs have reported relapse rates of 22% per year [3]. The subsequent episodes seem to worsen the prognosis and increase the suicide risk [4,5]. Patients in the initial phases of BD appear to better respond to treatment; thus, early intervention strategies could be vital for improving illness outcomes [6] by reducing conversion rates to full-blown illness and reducing symptom severity.

Mobile apps allow for real-time collection of both self-reported and objective data on behavioral activities or speech in naturalistic settings [7-12]. Active monitoring through self-reported data was found to correlate with scores on the depression (Hamilton Depression Rating Scale [HDRS]) and mania scales (Young Mania Rating Scale [YMRS]) [7,8,12]. As there are disturbances in diurnal rhythms and daily life regularity of patients with BD [13], continuous self-monitoring might be helpful for managing daily activities. Moreover, self-assessment data can be easily shared with mental health professionals and facilitate making clinical decisions [8,14]. Nevertheless, such monitoring could be less reliable in case of hypomanic/manic symptoms owing to decreased illness insight [12]. The other limitation for certain patients might be the necessity to respond to daily surveys [15]. It has not been also proven so far that such self-monitoring could predict phase change [8].

Data collected through passive objective monitoring of behavioral activities such as phone call statistics, data on physical activity and mobility, or voice features are correlated with scores on the HDRS and YMRS [8-11]. They could serve as markers for monitoring illness activity; thus, behavioral tracking is very promising in recognizing and predicting mood state [12]. Passively collected data do not require any involvement of the patient and could be beneficial in the early detection of subthreshold symptoms of episode recurrence. Disadvantages of passive behavioral tracking include privacy concerns, the discomfort of being observed, or problems related to the collection, analysis, and processing of data. Nevertheless, both active and passive monitoring through smartphone are promising for the assessment of illness activity in patients with BD.

Recent reviews by Antosik-Wójcińska et al [16], Rajagopalan et al [17], and Torous et al [18] agree that the medical potential of smartphone-based monitoring is high, but large-scale methodologically rigorous studies are necessary to draw well-generalizable conclusions. In another review, Rucci et al [19] argue that although nothing can substitute the clinical assessment, smartphone-based monitoring has the potential of improving the treatment owing to its accessibility to patients and the possibility of continuous tracking parameters reflecting illness activity. This could be of great importance, as the frequency of routine assessments during follow-up visits is often insufficient and the latency in identifying the recurrence of a mood episode is often long—3 weeks or more [20]. Consequently, adjustment of the treatment often takes place late—sometimes during a full episode [21]. This is particularly relevant in hypomanic/manic episodes because manic patients, often owing to decreased insight, do not seek medical help [20,22]. Notably, patients with BD are generally open to using smartphones to help them monitor their mental state [23,24]. The usefulness and ease of use of such apps were found to influence patients’ satisfaction and adherence [25,26]. Nevertheless, numerous challenges exist, especially concerning safeguarding privacy and ensuring data security [27].

In this paper, we report the results of a prospective observational study of patients with BD. The main goal was to assess whether behavioral data about smartphone usage collected automatically via a dedicated app, called BDmon, correlate with the severity of symptoms on both the HDRS and the YMRS and with the corresponding affective states in BD. Evaluation of the effect size and its statistical significance was based on generalized linear models with random effects for each patient. The secondary objective was to evaluate the suitability, in terms of completeness, of self-assessment data collected via the app over time in naturalistic settings.

## Methods

### Study Participants

This study was conducted in the Department of Affective Disorders, Institute of Psychiatry and Neurology in Warsaw and in a center specializing in clinical trials between September 2017 and December 2018. Patients were enrolled from both inpatient and outpatient settings to capture both mild and severe episodes. Based on previous studies [28,29], it was expected that patients would change the phase at least once in the period envisaged for this study. This study obtained the consent of the Bioethical Commission at the District Medical Chamber in Warsaw (agreement KB/1094/17).

We used the following inclusion criteria: adults aged 18 years or older, who gave their informed written consent to participate in this study, diagnosed with BD, with at least two changes of phase in the last 12 months, and using a smartphone with internet access daily or declaring their willingness to use it for the study period. Exclusion criteria consisted of serious hearing problems and speech disorders (eg, dysarthria, aphasia).

### Sample Size

The resulting sample included 84 eligible patients diagnosed with BD (according to the International Classification of Diseases, tenth revision [ICD-10]). The baseline sociodemographic and clinical characteristics of the final study sample are presented in Table 1.

**Table 1:** Sociodemographic and clinical characteristics of the final study sample (N=51).

Characteristics		Values
Participation time (days), mean (SD)		208 (32)
Age (years), mean (SD)		36.2 (9.5)
**Gender, n (%)**		
	Female	28 (55)
	Male	23 (45)
**Demographic living status, n (%)**		
	Family	30 (59)
	Partner	9 (18)
	Self	12 (24)
**Education, n (%)**		
	Elementary	2 (4)
	Secondary	18 (35)
	Higher	31 (61)
**Demographic residence, n (%)**		
	City	37 (73)
	Town	11 (22)
	Village	3 (6)
**Occupation, n (%)**		
	Working	29 (57)
	Pensioner	11 (22)
	Student	6 (12)
	Other	5 (9)
**Clinical characteristics**		
	Mean duration of illness (years), mean (SD)	7.1 (5.3)
	Hospitalizations, median (IQR)	2 (0-7)
	Affective episodes, median (IQR)	6 (2-10)
	Bipolar disorder type I, n (%)	31 (61)
	Bipolar disorder type II, n (%)	20 (39)

### Psychiatric Assessment

The assessment of the mental state was carried out by psychiatrists with experience in the diagnosis and treatment of BD. Both the researcher and the patient were blinded to the data automatically collected by the BDmon app. Patients were invited to visit the researcher at least every 3 months. In the case of a suspected change in the mood state, patients were invited for an additional intervention visit. During personal visits, the primary outcome measures (17-point version of HDRS and YMRS) were used. In this paper, the assessment of HDRS and YMRS is used as binding to assess the severity of symptoms and the corresponding BD states.

The data concerning clinical and demographic characteristics were collected as a part of the initial visit. During the following visits, the patients were inquired about significant changes in the general health status and other important life events that might affect their activity and behavior. The patient’s mood state was also assessed using fortnightly phone-based interactions with the researcher. The basic and the most important objective of the phone-based assessment was to determine whether any significant change in the mood state was likely to occur since the previous contact with the patient. If so, the patient was invited for an intervention visit, and a full assessment using HDRS and YMRS was conducted. To shorten the time of the fortnightly calls, the researcher read to the patient questions prepared in the telephone questionnaire form (see Multimedia Appendix 1).

Based on HDRS and YMRS, the mood state was classified into 1 of the 4 phases (depression, hypomania/mania, euthymia, or mixed state). In previous studies, researchers have adopted different cutoff points on the HDRS and YMRS for training classifiers [11,12,30]. High thresholds of 13 points on the HDRS and YMRS [12] increase the validity of the diagnosis, focusing on more severe symptoms. The consequence of this approach might be counting the milder symptoms of a given phase to euthymia. We aimed at the identification of early symptoms of phase change and hence adopted lower cutoff points. The state of euthymia was defined as HDRS<8 and YMRS<6, depression as HDRS≥8 and YMRS<6, hypomania/mania as HDRS<8 and YMRS≥6, and mixed state as HDRS≥8 and YMRS≥6.

Smartphones collect behavioral data continuously whereas clinical assessments are much less frequent. Supervised learning requires both data types to be present. Consequently, we extrapolated the psychiatric assessment to 7 days before and 2 days after a visit. In the final sample, we included only patients with automatically collected smartphone data within this period. The asymmetry of the time window was inspired by Muaremi et al [31] who argue that a visit is typically related to some medical intervention, which might lead to a change in the patient’s mental state. The time frame before the visit differs across studies, for example, Faurholt-Jepsen et al [11,12] adopted a time span of the clinical assessment days and 3 previous days. In this study, we extended this period up to 7 days before the visit because we had additional data about patient’s mental state (derived from phone-based visit and from patients’ relatives). Nevertheless, to facilitate meta-analyses, we additionally performed the analysis for the shorter period assuming ground truth of 3 days before the psychiatric assessment and the day of psychiatric assessment, as well for different cutoff points (Multimedia Appendix 2 and Multimedia Appendix 3).

### Smartphone-Based Data Collection

The BDmon app requires a smartphone with the Android system, which at the time of the clinical trial had over 90% of the market share in Poland. Patients used their own smartphones or were offered a new smartphone for the study period. Patients did not receive any financial gratification for participation in the study. The BDmon app collected 3 groups of data:

(1) objective data about patient’s behavior related to smartphone (statistics about phone calls and text messages), (2) self-reported data completed by patients via the app (filling in the questionnaires was not mandatory; Multimedia Appendix 4), and (3) objective acoustic features about patient’s speech extracted from his/her daily phone calls. Only self-assessment (2) required the patients to interact with the software; data from groups (1) and (3) were collected passively. The scope of monitoring was as follows:

1. Objective data about patient’s behavior consisted of statistics of calls and text messages transformed into 11 daily aggregates describing behavioral parameters. They fall into 4 categories: (1) incoming answered calls: number (per day), mean duration (seconds/call), and variability of duration (the standard deviation of the lengths of all calls/day; seconds); (2) incoming missed calls: number (per day) and fraction of missed calls; (3) outgoing calls: number (per day), mean duration (seconds/call), variability of duration (standard deviation/day; seconds), and fraction of outgoing calls (ratio of the number of outgoing calls to the sum of outgoing and incoming calls); and (4) outgoing text messages: number (per day) and average number of characters in outgoing messages (per day). Initially, the app collected data concerning mobility (daily travelled distance) and activity (pedometer), but owing to the technical issues and patients’ privacy concerns, these data were mostly missing and were not eventually analyzed.
2. Self-reported data completed by patients via the app: filling in the self-assessment questionnaires was not mandatory to evaluate, in natural settings, their completeness over time, and relevance for analysis. Patients assessed their well-being using a dedicated mood rating scale created for the purposes of the study. Each patient had access to a graph illustrating the mood status and the length of sleep.
3. Acoustic features of patient’s speech signal consisting of 85 physical parameters describing each phone call: acoustic features require dedicated processing, which is beyond the scope of this paper. The preliminary analysis of the acoustic data is a subject of other ongoing and completed works [32,33].

### Statistical Methods

To assess the strength of the relation between the behavioral markers and the affective symptoms and BD states, we applied generalized linear mixed-effects models similarly as in the work of Faurholt-Jepsen et al [12]. First, mixed-effects linear regression was applied with scores on either HDRS or YMRS as response variables. This quantifies the correlations between behavioral markers and the severity of affective symptoms. Second, mixed-effects logistic regression was used for binary classification discriminating between an affective state and euthymia to describe the relation between the behavioral markers and affective states. The generalized linear mixed-effects model had the following form:

*y* = *Xβ* + *Zu* + *ε*

where *y* is an *n*_d_ × 1 vector consisting of *n*_d_ responses, each of which corresponds to 1 patient and 1 day. When assessing the severity of symptoms, either the HDRS or YMRS scores are used as the response vectors.

*X* is an *n*_d_ × *s* covariate matrix of the *s* predictor variables for fixed effects *β* (regression coefficient). The fixed effects consist of coefficients related to the objective smartphone data (eg, number of incoming calls) and a constant term. We build a separate model for each of the predictor variables; therefore, *s*=1.

*Z* is the *n*_d_ × *q* covariate matrix for the random effects *u*. The random effects occur at the patient level (level two) and are patient-specific random intercepts. Consequently, *q* is equal to the number of patients (groups) for whom data were available.

*ε* is the vector of *n*_d_ errors, which is assumed to be multivariate normal with zero mean. The number of patients differs depending on the considered parameter. For example, if patient A was never sending text messages, the daily number of sent text messages was calculated as 0. This allowed us to include patient A for the assessment of the relation between symptoms and the number of sent text messages. At the same time, sending no text messages led to missing values reported for the mean number of characters of sent text messages, and thus, patient A was not counted for assessment of the relation between symptoms and the length of sent text messages. Logistic mixed-effects regression models were applied to assess the relations between pairs of euthymia and the affective states (eg, euthymia vs depression). For each of the logistic models, the response variable equaled 0 for days with the euthymic state and 1 for days with the affective state (eg, depression). The inverse of the log link binomial function *h*(*η*) *=* (1+exp(*η*))^−1^ was applied to the linear predictor *η=Xβ + Zu* to relate it to the outcome *y*. The mixed-effects logistic regression model was formulated as follows:

*y* = *h*(*Xβ* + *Zu*) + *ε*

Model assumptions were checked with residual analysis and visually using quantile-quantile plots. The *P* values were calculated assuming normal distributions of errors. However, for some variables, especially the number of outgoing and incoming calls per day, this assumption seemed to be violated. Therefore, caution is necessary when interpreting the *P* values of the mixed-effects models; see also Luke [34]. Log transformations were applied to minimize this effect and the results were gathered for comparative purposes in Multimedia Appendix 5 and Multimedia Appendix 6. All analyses were conducted in the R programming language (R Core Team and the R Foundation for Statistical Computing). Linear and logistic mixed-effects models were calculated using packages lme (for the restricted maximum likelihood estimation) and lmerTest (*P* values and model diagnostics) available at the Comprehensive R Archive Network repository for R language [35]. We set the significance level to .05.

## Results

### Study Sample

**Figure 1:**
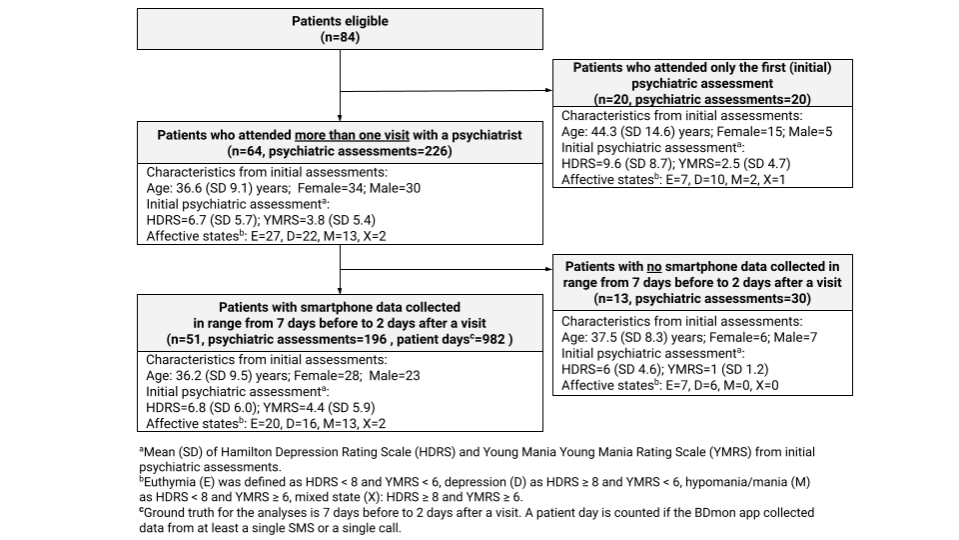
Flow chart illustrating the number of patients, psychiatric assessments, and patient days during the study.

Figure 1 illustrates the participant flow in this study; 84 eligible patients diagnosed with BD (according to ICD-10 classification) were enrolled in this study, and they participated in the initial interview with the psychiatrist. Disregarding their declarations of participation in this study, 20 patients dropped out after the initial psychiatric assessment. As depicted in Figure 1, the mean age (36.6 [SD 9.1] years) was lower for the group of 64 patients who continued the study in comparison to that of the group of 20 patients who resigned after the initial visit (44.3 [SD 14.6] years). For the remaining 64 patients, there were 226 psychiatric assessments in total. However, smartphone-based automatically collected behavioral data in the assumed time frame (7 days before and 2 days after psychiatric assessment) were available for only 51 patients (196 psychiatric assessments). These patients (N=51) were considered as the final study sample. In total, this constitutes 982 patient days with data for the statistical analyses. The mean participation time for the final sample of 51 patients, calculated as the difference between the initial and the last psychiatric assessment, was 208 days with a standard deviation of 132 days.

### Psychiatric Assessments and Affective States

Overall, 196 psychiatric assessments were reported for the final study sample. We observed 145 mood state transitions among consecutive psychiatric interviews as summarized in Table 2.

Out of 145 transitions, in 74 (51%) cases, the affective state changed from the previous one and in 71 (48.9%) remained the same. The most frequent change was from euthymia to depression state (17 such cases) and from depression to euthymia (14 cases).

**Table 2:** Transitions among the affective states for consecutive visits for patients of this study (N=51).

Flow from the following phases	To the following phases			
	Euthymia	Depression	Mixed state	Mania
Euthymia	25	17	2	6
Depression	14	39	4	4
Mixed state	4	2	1	2
Mania	9	5	5	6

### Completeness of Data Collected From Smartphones

**Figure 2:**
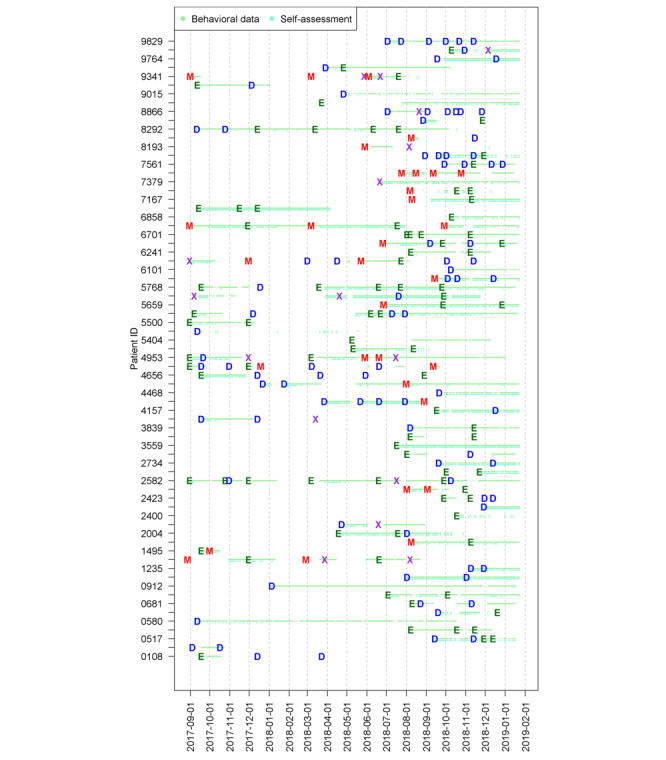
Completeness of data: for each patient, the green dots mean that behavioral data were collected, turquoise dots represent self-assessment data, and letters denote the clinically assessed affective state of patients with bipolar disorder. D: depression; E: euthymia; M: mania; X: mixed.

Figure 2 illustrates the completeness of the objective behavioral data collected from smartphones. For each patient, there are 2 rows of dots. Green dots in the upper row show days, in which the objective behavioral data were collected. Turquoise dots in the lower row indicate days with self-assessment data. Less frequent usage of the app in early 2018 was a result of technical stability issues with the app.

Overall, we identified 982 person days with smartphone data and psychiatric assessments. The summary statistics of behavioral and self-assessment data are presented in Table 3 for all variables as initially planned for this study.

**Table 3:** Basic summary statistics of the behavioral and self-assessment data collected from smartphones.

Type, daily variable		Completeness, n (%)	Median		Mean (SD)	
**Phone calls (count per patient day)**						
	Number of incoming answered calls	982 (100)		2.0		3.2 (3.5)
	Mean duration of incoming calls (s/call)	820 (83.5)		119.9		210.3 (286.3)
	SD of duration of incoming calls (s)	600 (61.1)		117.7		234.5 (324.3)
	Number of outgoing calls	982 (100)		4.0		7.5 (10.9)
	Mean duration of outgoing calls (s/call)	886 (90.2)		79.1		147.2 (232.8)
	SD of duration of outgoing calls (s)	756 (77.4)		113.3		195.9 (245.7)
	Fraction of outgoing calls	886 (90.2)		0.7		0.7 (0.2)
	Number of missed calls	982 (100)		1.0		1.8 (2.7)
	Fraction of missed calls	982 (100)		0.2		0.2 (0.2)
	Number of incoming + outgoing calls	963 (98.0)		105.8		170.2 (229.4)
	Mean duration of incoming + outgoing calls (s/call)	877 (89.3)		145.8		234.0 (256.8)
	SD of duration of incoming + outgoing calls (s)	982 (100)		8.0		12.5 (14.4)
**Text messages**						
	Number of sent text messages	982 (100)		0.0		3.8 (13.3)
	Mean length of text messages	343 (34.9)		39.4		54.3 (48.7)
**Activity**						
	Sum of steps	103 (10.5)		480		5299.9 (32454.8)
	Daily travelled distance (km)	—^a^		—		—
**Self-assessment**						
	Self-assessment of sleep time (h)	270 (27.5)		8.0		8.0 (2.5)
	Self-assessment of mood (from −4 to +4)	268 (27.3)		0.0		−0.6 (1.6)
	Comment about sleep (number of characters)	39 (4)		22.0		27.1 (21.0)
	Comment about mood (number of characters)	37 (4)		25.0		40.2 (46.6)

^a^Not collected due to technical issues and privacy concerns.

The app was designed to collect data concerning mobility (daily travelled distance) and the activity measures with the number of steps (pedometer), but owing to the technical issues and patients’ privacy concerns, the completeness of these data (98/982, 9.9%) was insufficient to conduct a reliable analysis. Similarly, the collected self-reported comments about sleep and mood were characterized by relatively high rates of missing data (n=39 and n=37, respectively). Table 4 provides the sociodemographic and clinical characteristics of patients depending on their adherence in terms of filling in self-assessment questionnaires. The study patients were split into 3 groups depending on the completeness of the self-assessment data; 16 patients out of 51 patients (31%) demonstrated low adherence to filling in self-assessment questionnaires (completeness less than 98 patient days). The group of patients with low adherence had relatively high sum of points on the HDRS (7.25 [SD 5.05]) and low sum of points on the YMRS (1.81 [SD 1.64]). Interestingly, 10 patients out of 16 in the group with low adherence (62.5%) were assessed as euthymic during the initial assessment.

**Table 4:** Sociodemographic and clinical characteristics of the patients grouped according to their adherence of filling the self-assessment questionnaires (N=51).

Characteristics		Data completeness of filling the self-assessment		
		Low (<10%)	Medium (10%-90%)	High (>90%)
Group of patients according to adherence of filling the self-assessment, n (%)		16 (31)	29 (57)	6 (12)
**Gender, n (%)**				
	Female	7 (14)	18 (35)	3 (6)
	Male	9 (18)	11 (22)	3 (6)
**Demographic living status, n (%)**				
	Family	11 (22)	13 (25)	6 (12)
	Partner	2 (4)	7 (14)	0
	Self	3 (6)	9 (18)	0
**Education, n (%)**				
	Elementary	0	2 (4)	0
	Secondary	4 (8)	10 (20)	4 (8)
	Higher	12 (24)	17 (33)	2 (4)
**Demographic residence, n (%)**				
	City	11 (22)	22 (43)	4 (8)
	Town	4 (8)	6 (12)	1 (2)
	Village	1 (2)	1 (2)	1 (2)
Age (years), mean (SD)		37.31 (8.66)	36.41 (8.69)	32.67 (15.28)
**Severity of symptoms from the initial psychiatric assessment, mean (SD)**				
	Hamilton Depression Rating Scale	7.25 (5.02)	6.97 (6.89)	5 (4.15)
	Young Mania Rating Scale	1.81 (1.64)	5.48 (6.35)	6.5 (8.65)
**Affective state assessed during initial psychiatric assessment (n)**				
	Euthymia	10	7	3
	Depression	6	9	1
	Mania	0	11	2
	Mixed state	0	2	0

### Between-Patient and Intrapatient Variability

**Figure 3:**
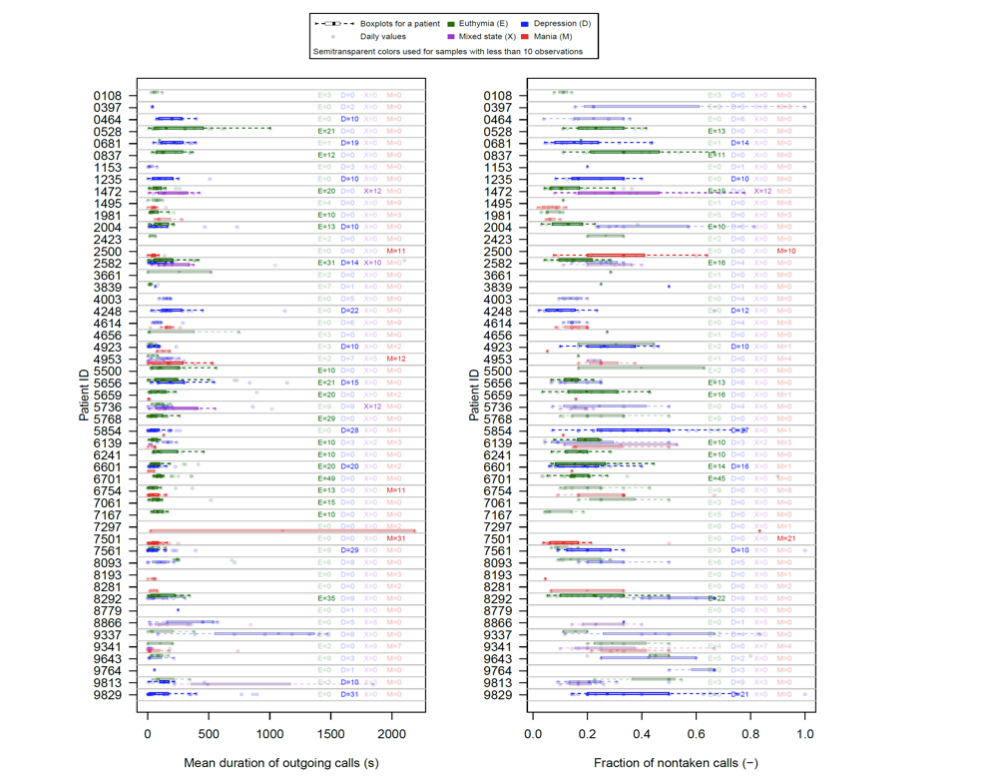
Intrapatient variability of 2 objective parameters collected with smartphones among chosen patients with bipolar disorder.

The summary statistics about the behavioral and self-assessment data in various affective states are shown in Multimedia Appendix 7. For most of the considered variables, differences in statistics were observed between the 4 groups (euthymia, depression, mania, mixed state). Apart from the overall difference of statistics for patients in various BD states, there was also a relatively high intrapatient variability in phone call statistics. Figure 3 illustrates this for 2 objective parameters and all patients with BD. The boxplots for all the variables are presented in Multimedia Appendices 8-19.

### Relation Between Smartphone-Based Data and Severity of Depressive and Manic Symptoms

Table 5 presents the estimates of the regression coefficients *β.* They describe the influence of behavioral data on the severity of manic and depressive symptoms measured by the number of HDRS and YMRS points. The number of observations is the product of the number of groups (ie, patients) and days for which the considered data type was collected.

The results show that the more severe the manic symptoms: (1) the higher variability in the duration of incoming calls (*P*=.04) and outgoing calls (*P*=.02), (2) the higher number and fraction of missed calls (*P*=.02), (3) the higher number of outgoing text messages (*P*<.001), (4) the higher number of characters in outgoing text messages (*P*<.001), and (5) the higher score on self-reported mood rating (*P*<.001). For example, for every increase of 1 missed call, there was an increase of 0.141 points on the YMRS. More severe depressive symptoms are associated with (1) lower number of incoming answered calls (*P*<.001), (2) higher fraction of incoming missed calls (*P*<.001), and (3) lower score on self-reported mood rating (*P*<.001).

**Table 5:** Relations between smartphone-based data collected using the BDmon app and depressive and manic symptoms assessed with the Hamilton Depression Rating Scale and Young Mania Rating Scale, respectively.

Daily variable	Depressive symptoms to			Manic symptoms			Patient days (n)	Patients (n)
	Regression coefficient (β)	*P* value	95% CI	Regression coefficient (β)	*P* value	95% CI		
Number of incoming answered calls	−.149	<.001	−0.231 to −0.066	−.079	.10	−0.174 to 0.016	982	51
Duration of incoming calls (s/call)	.001	.17	0 to 0.002	.001	.22	0 to 0.002	820	50
Standard deviation of incoming calls duration (s)	.001	.29	0 to 0.001	.0012	.04	0 to 0.002	600	48
Number of outgoing calls	−.023	.09	−0.05 to 0.004	.002	.90	−0.029 to 0.033	982	51
Fraction of outgoing calls	.307	.64	−0.994 to 1.604	.985	.19	−0.492 to 2.471	886	51
Duration of outgoing calls (s/call)	.001	.37	−0.001 to 0.002	.001	.09	0 to 0.002	886	51
Standard deviation of outgoing calls duration (s)	0	.91	−0.001 to 0.001	.0017	.02	0 to 0.003	756	48
Number of missed calls	.06	.27	−0.047 to 0.167	.141	.02	0.019 to 0.263	982	51
Fraction of missed calls	4.861	<.001	2.829 to 6.896	2.991	.02	0.434 to 5.526	582	50
Number of sent text messages	.02	.06	−0.001 to 0.041	.116	<.001	0.093 to 0.139	982	51
Mean length of text messages (number of characters)	.004	.30	−0.003 to 0.011	.022	<.001	0.011 to 0.034	343	38
Self-assessment of sleep time (h)	−.027	.83	−0.274 to 0.22	−.130	.19	−0.325 to 0.062	270	39
Self-assessment of mood	−1.452	<.001	−1.777 to −1.125	.509	<.001	0.267 to 0.751	268	42

### Relation Between Smartphone Data and Affective States

Tables 6 and 7 present the results from generalized mixed regression models (logistic model). Smartphone-based data were used as predictors. We were interested in the detection of behavioral changes between euthymia, which we treated as a reference and encode as 0, and the other affective states encoded as 1. Therefore, for each predictor, we fitted 4 regression models distinguishing euthymia from depression, mania, mixed state as well as a combination of manic and mixed states. Positive values of the regression coefficients indicate that the predictor tends to have lower value in the reference euthymic state. As we put emphasis on the early identification of phase change by adapting lower cutoff points on the above scales, we were able to distinguish between euthymia and other affective states, both mild and severe, that is, depression, hypomania/mania, or mixed state.

**Table 6:** Mixed regression models regarding behavioral smartphone-based data and affective states (euthymia, depression, and mania) in patients with bipolar disorder assessed with the Hamilton Depression Rating Scale and Young Mania Rating Scale.

Daily variable	Euthymia versus depression					Euthymia versus mania				
	Regression coefficient (β)	*P* value	95% CI	Observations (n)	Patients (n)	Regression coefficient (β)	*P* value	95% CI	Patient days (n)	Patients (n)
Number of incoming answered calls	−.15	.01	−0.243 to −0.056	789	46	.024	.71	−0.103 to 0.152	567	41
Duration of incoming calls (s/call)	0	.41	0 to 0.001	664	46	−.001	.59	−0.004 to 0.002	488	39
Standard deviation of duration of incoming calls (s)	0	.45	−0.001 to 0.001	490	44	−.001	.41	−0.005 to 0.002	375	36
Number of outgoing calls	−.064	.01	−0.113 to −0.014	789	46	.044	.16	−0.018 to 0.106	567	41
Fraction of outgoing calls	1	.09	−0.151 to 2.152	715	46	2.73	.03	0.223 to 5.237	526	41
Duration of outgoing calls (s)	.001	.31	−0.001 to 0.002	715	46	0	.88	−0.003 to 0.003	526	41
Standard deviation of duration of outgoing calls (s)	0	.95	−0.001 to 0.001	603	42	−.002	.31	−0.006 to 0.002	463	41
Number of missed calls	−.017	.77	−0.132 to 0.098	789	46	−.03	.76	−0.227 to 0.167	567	41
Fraction of missed calls	4.431	<.001	2.12 to 6.742	463	45	1.912	.38	−2.335 to 6.158	345	40
Number of sent text messages	.001	.94	−0.036 to 0.039	789	46	.015	.69	−0.06 to 0.09	567	41
Mean length of text messages (number of characters)	.001	.91	−0.016 to 0.018	265	28	.014	.08	−0.002 to 0.029	192	28
Self-assessment of sleep time (h)	−.254	.08	−0.538 to 0.03	219	33	−.548	.19	−1.369 to 0.273	147	30
Self-assessment of mood	−2.056	<.001	−2.058 to −2.055	224	35	.676	.22	−0.406 to 1.758	144	31

**Table 7:** Mixed regression models regarding smartphone-based data and affective states (euthymia, mixed/manic states) in patients with bipolar disorder assessed with the Hamilton Depression Rating Scale and Young Mania Rating Scale.

Daily variable	Euthymia versus mixed state					Euthymia versus mania and mixed states				
	Regression coefficient (β)	*P* value	95% CI	Observations (n)	Patients (n)	Regression coefficient (β)	*P* value	95% CI	Patient days (n)	Patients (n)
Number of incoming answered calls	−.114	.08	−0.244 to 0.015	516	35	−.043	.32	−0.127 to 0.042	638	42
Duration of incoming calls (s/call)	.002	.06	0 to 0.004	448	34	.001	.26	−0.001 to 0.002	546	40
Standard deviation of duration of incoming calls (s)	.0027	.02	0 to 0.005	339	33	.001	.11	0 to 0.003	412	38
Number of outgoing calls	−.035	.13	−0.081 to 0.01	516	35	.005	.64	−0.016 to 0.026	638	42
Fraction of outgoing calls	.44	.70	−1.799 to 2.68	477	35	1.363	.11	−0.318 to 3.045	587	42
Duration of outgoing calls (s)	.0015	.045	0 to 0.003	477	35	.001	.06	0 to 0.002	587	42
Standard deviation of duration of outgoing calls (s)	.0031	.01	0.001 to 0.005	420	35	.0012	.045	0 to 0.002	518	42
Number of missed calls	.135	.07	−0.012 to 0.282	516	35	.08	.18	−0.036 to 0.195	638	42
Fraction of missed calls	4.928	<.001	4.926 to 4.93	310	35	3.53	.01	0.907 to 6.153	387	42
Number of sent text messages	.032	.01	0.009 to 0.055	516	35	.031	.01	0.009 to 0.052	638	42
Mean length of text messages (number of characters)	.014	.07	−0.001 to 0.029	180	21	.015	.01	0.003 to 0.026	225	29
Self-assessment of sleep time (h)	−.121	.62	−0.606 to 0.363	144	24	−.334	.08	−0.708 to 0.04	171	31
Self-assessment of mood	.166	.69	−0.666 to 0.998	142	25	.348	.31	−0.327 to 1.022	165	32

The following variables discriminate between euthymia and depression: (1) number of incoming answered calls (*P*=.01), (2) fraction of missed calls (*P*<.001), and (3) number of outgoing calls (*P*=.01). Euthymia and mania differ significantly in fraction of outgoing calls (*P*=.03). The mixed states observed in this study were mainly mixed manic states, which was reflected in the average scores on HDRS (10.6 [SD 2.9]) and YMRS (17.6 [SD 10.2]). This is the rationale behind conducting additional analysis to catch the whole spectrum of manic or mixed features by combining both states into 1 group in Table 7. The following variables turned out to be relevant and discriminate the above conditions from euthymia: (1) fraction of missed calls (*P*=.01), (2) variability of the duration of calls (*P*=.045), (3) number of sent text messages (*P*=.01), and (4) mean length of text messages (*P*=.01).

Further, in Table 8, we discriminate euthymia from the pathological states considered as 1 group to find out which predictors are relevant markers for BD. The following 7 out of 13 variables (54%) are statistically significant: (1) number of incoming calls (*P*=.002), (2) fraction of outgoing calls (*P*=.01), (3) fraction of missed calls (*P*<.001), (4) number of sent text messages (*P*=.01), (5) mean length of text messages (*P*=.01), (6) self-reported sleep time (*P*=.03), and (7) self-reported mood (*P*=.003). It needs to be noted that 6 out of these 7 variables (all except for the self-reported sleep time) are statistically significant and discriminate euthymia from the pathological states considered as 1 group also for the longer period (14 days) preceding the psychiatric assessments (see Multimedia Appendices 8-20).

**Table 8:** Mixed regression models regarding smartphone-based data and affective states (euthymia vs depressive/mixed/manic states) in patients with bipolar disorder assessed with the Hamilton Depression Rating Scale and Young Mania Rating Scale.

Daily variable	Euthymia versus depression/mania/mixed state				
	Regression coefficient (β)	*P* value	95% CI	Observations (n)	Patients (n)
Number of incoming answered calls	–.101	.002	–0.164 to –0.038	982	51
Duration of incoming calls (s/call)	.000	.29	0 to 0.001	820	50
Standard deviation of duration of incoming calls (s)	.001	.24	0 to 0.001	600	48
Number of outgoing calls	–.005	.55	–0.022 to 0.012	982	51
Fraction of outgoing calls	1.211	.01	0.244 to 2.179	886	51
Duration of outgoing calls (s)	.001	.07	0 to 0.002	886	51
Standard deviation of duration of outgoing calls (s)	.0004	.34	0 to 0.001	756	48
Number of missed calls	.0496	.24	–0.033 to 0.132	982	51
Fraction of missed calls	4.494	<.001	4.492 to 4.496	582	50
Number of sent text messages	.023	.01	0.006 to 0.041	982	51
Mean length of text messages (number of characters)	.010	.04	0 to 0.02	343	38
Self-assessment of sleep time (h)	–.261	.03	–0.493 to –0.03	270	39
Self-assessment of mood	–.551	.003	–0.913 to –0.189	268	42

### Summary of the Analyses

The most clinically relevant data that could be drawn from both presented analysis types are as follows: (1) number of incoming answered calls was lower in patients with depression as compared to those with euthymia and, at the same time, missed incoming calls were more frequent and increased as depressive symptoms intensified; (2) depressed patients tended to make phone calls less frequently than euthymic patients; (3) fraction of missed calls was higher in manic/mixed states and was positively correlated to manic symptoms; (4) fraction of outgoing calls was higher in manic states; (5) variability of duration of calls was higher in manic/mixed states and positively correlated to the severity of symptoms; and (6) the number and length of sent text messages was higher in manic/mixed states as compared to euthymic state and positively correlated to the severity of manic symptoms. The self-reported mood scores were significantly correlated with depressive and manic symptoms (as measured with the HDRS and YMRS), but data were insufficient to explain their relation to BD states.

## Discussion

### Principal Findings

To the best of our knowledge, this is one of the largest studies investigating the relation between the BD phase assessed by psychiatrists and the objective behavioral data collected via smartphones. Reliable smartphone-based results were obtained for 51 patients. This paper confirms and further extends the findings presented by Faurholt-Jepsen et al [12] concerning objective behavioral markers based on phone call statistics. Our results confirm that the majority of phone call statistics are significantly correlated with the clinically rated depressive and manic symptoms (assessed using the HDRS and YMRS, respectively) and are valid markers distinguishing between BD phases (mania, depression, mixed state, euthymia). We observed that depressed patients made phone calls less frequently than euthymic patients; they also answered the phone less often, and this behavior increased as the depressive symptoms intensified. However, patients in the manic/mixed states used phones more frequently than patients in the euthymic state, which was seen in the higher fraction of outgoing calls and a higher number and length of text messages. In general, these results are consistent with those reported in a previous research. For example, in the studies of Faurholt-Jepsen et al [8] and Muaremi et al [31], the number and length of phone calls were correlated with the BD phase and were found to be higher in mania and lower in depression. However, the available studies are not entirely homogeneous in terms of the given phone call parameter and its direction of changes in the particular phase of BD [8,12,31,36]. Patients present different behavioral patterns depending on individual characteristics and preferences for using a mobile phone. We suppose that this issue may at least partly depend on the analyzed group of patients and intrapatient variability. In our study, we also observed that patients in manic/mixed states demonstrated various phone usage patterns—from very long calls to very rare phone use. This was especially reflected in the large standard deviation of the duration of calls. To our knowledge, this variability has not been investigated in detail in similar studies so far. This could open up an interesting field for further research focused on tailored solutions for the individual patient rather than on the characteristics of BD itself. It seems that taking precautions in considering call statistics as a single-phase marker or phase change marker for all patients, in particular, in manic/mixed state, would be reasonable. The behavioral parameters differ between mood phases (depression, mania, mixed state) as compared to euthymia. Depression and mania reflect various manifestations of the illness, but not necessarily should be seen as two poles of the same dimension [37]. Consequently, parameters characterizing depression and mania do not need to have opposite signs but will rather be different from euthymic state. In our study, this conclusion was also confirmed by the observation of missed calls among patients with depression and mania or mixed state. This parameter was significantly increased in both affective states as compared to euthymia and was positively correlated to the severity of depressive and manic symptoms. This could indicate that both manifestations of the disease, although of different affective tones, have a similar effect on social interactions, and its size increases with the severity of the illness symptoms. In depression, this could be due to psychomotor retardation and social isolation, while in manic/mixed states, this could be the effect of growing distractibility and inattention. Therefore, we conjecture that the key information might be conveyed by the general change in behavioral patterns appropriate for a given patient in euthymia, and not the direction of the change itself. The above conclusion is also supported by an analysis comparing all pathological affective states in BD to the euthymic state (Table 8), showing a significant difference with the euthymic state. The correlation of self-assessment data with the clinically rated depressive or manic symptoms has been shown in the previous research [8,38-41]. It is worth noting at this point that there are also apps providing personalized psychoeducation programs or mobile therapy combined with self-assessment tools, for instance, the SIMPLe app [42]. The SIMPLe app showed efficacy in improving sleep, social rhythms, and eating pattern [43].This paper also revealed that in natural settings, patients’ adherence declined over time. We observed that at the very beginning, almost 90% of the patients filled data regularly, but after 3 months, only less than one-quarter of the patients systematically continued the self-assessment. This adherence rate appeared to be similar to that reported by Hidalgo-Mazzei et al [44] where only 30% of the patients were using the app regularly after 6 months of the study. Nevertheless, it should be stressed that adherence in completing self-rated questionnaires ranging from 42% to 95% was observed in previous studies [45]. We have identified that most of the patients with low adherence in filling self-assessment questionnaires were in the state of euthymia during the initial psychiatric assessment. The other hypothesis is that cognitive performance might play a certain role in filling the assessments [46]; yet, the data collected in this study provides little information on this topic. Further research might take this potential variable into consideration. The long-term collection of self-assessment data and their suitability for the recognition and prediction of affective state seem to be problematic. Similar conclusions were also drawn by Faurholt-Jepsen et al [8]. The completeness of the self-assessment data collected in this study was insufficient to fully explain their relation to BD states. A strength of this study is that most patients were assessed several times using a longitudinal design for an average of ~7 months. The adopted cutoff points on the depressive (HDRS) and manic (YMRS) scales allowed us to capture changes in phone usage patterns that are already different in mild depression/hypomania/mania/mixed states as compared to euthymia. Patients with severe manic/mixed symptoms also tended to provide more diversified patterns of behavior in the same condition. This sheds new light on, so far little studied, the variability of behavior among patients with BD (measured for example with the standard deviation) in given affective states. Further research could concentrate on personalized apps, adapting to a given patient, or the search for more generalizable smartphone-based objective parameters, which will be independent of the individual patterns of the phone usage and behavior. Acoustic features of the human voice seem to be promising candidates [47]. In the study of Faurholt-Jepsen et al [11], voice data turned out to be a reliable objective phase marker in BD. Moreover, speech features seem to be a promising marker for the assessment of suicide risk in patients with depression [48]. In our study, we also collected these data, but owing to their extensive nature and the need for thorough analysis, they will be presented in a separate paper. Relations between data collected from smartphones and the depressive and manic affective symptoms confirmed in this study demonstrate promising potential for both early detection of affective states and the prediction of phase changes. This is commonly formulated as either classification or regression task [49]. Recent papers [34,50] show that statistical and machine learning approaches can be complemented by process monitoring with control charts. These are easily understandable visual tools that naturally address the temporal structure of data and generate notifications about the change of BD phase.

### Limitations

The initially planned sample size was 100 patients. The reason for not meeting this goal was a slowdown in the trial in early 2018 due to technical issues (eg, monitoring of the daily travelled distance based on satellite navigation data, disruption of the normal usage of smartphone). However, it is worth noting that the final sample size (N=51) is still one of the largest among similar studies. The BDmon app was developed only for Android as it was the dominating operating system in Poland at the time of this study and we did not want to add additional costs and technical complexity to the project. During the enrolment into the study, only 3 out of 84 patients (4%) did not have an Android smartphone and were offered a relevant device for the study period. Therefore, we believe that the potential autoselection of our sample arising from this issue was negligible. The most controversial monitoring function for patients was travelled distance computed using satellite navigation—almost 90% of the patients refused to be monitored; thus, these data were not eventually analyzed. Moreover, patients in the manic state were likely to switch off the smartphone quite frequently or uninstall the app, which created nonrandom missing data. During this study, 3 patients experienced worsening of the psychotic symptoms accompanying depressive and manic episodes. Finally, since this study was exploratory, no correction for multiple comparisons was applied.

### Conclusions

This study brings strong evidence that smartphone-based parameters reflecting behavioral activities are related to the severity of depressive and manic symptoms and allow for discriminating between affective states in BD, that is, depression versus euthymia and manic/mixed states versus euthymia. These parameters could be used to assess the severity of manic or depressive symptoms in BD and assist in the early recognition of phase change, which can increase the patient’s chance of early intervention between outpatient visits and hence improve the course of the disease and prognosis.

## Appendix

Multimedia Appendix 1 Telephone questionnaire form.

Multimedia Appendix 2 Changed ground truth.

Multimedia Appendix 3 Changed cut-off points.

Multimedia Appendix 4 Well-being scale.

Multimedia Appendix 5 Correlations of phone calls.

Multimedia Appendix 6 Correlations of other calls.

Multimedia Appendix 7 Summary of statistics.

Multimedia Appendix 8 Boxplots of duration of all calls.

Multimedia Appendix 9 Boxplots of incoming calls.

Multimedia Appendix 10 Boxplots of outgoing calls.

Multimedia Appendix 11 Boxplots of all calls.

Multimedia Appendix 12 Boxplots of duration of incoming calls.

Multimedia Appendix 13 Boxplots of duration of outgoing calls.

Multimedia Appendix 14 Boxplots of fraction of missed calls.

Multimedia Appendix 15 Boxplots of standard deviations of incoming call duration.

Multimedia Appendix 16 Boxplots of standard deviations of outgoing call duration.

Multimedia Appendix 17 Boxplots of fraction of outgoing calls.

Multimedia Appendix 18 Boxplots of number of missed calls.

Multimedia Appendix 19 Boxplots of self-assessment of feeling, sleep time, number, and length of text messages.

Multimedia Appendix 20 Regression coefficients from mixed regression models regarding smartphone-based data and affective states.

## Abbreviations

BD: bipolar disorder
HDRS: Hamilton Depression Rating Scale
ICD-10: International Classification of Diseases, tenth revision
YMRS: Young Mania Rating Scale
