# Supplementary material for: Behavioral and Self-reported Data Collected From Smartphones for the Assessment of Depressive and Manic Symptoms in Patients With Bipolar Disorder: Prospective Observational Study
Source: J Med Internet Res. 2022 Jan 19;24(1):e28647. doi: 10.2196/28647 (PMC8811705; doi:10.2196/28647)
Supplement: Multimedia Appendix 1 [file jmir_v24i1e28647_app1.pdf]

Telephone Questionnaire Form for bipolar disorder patients
1. Have you recently experienced a change in mood?
2. Did people around you say they noticed your mood change?
3. Have you recently noticed a change of activity (energy level)?
4. Did people around you say they noticed a change in your activity (energy level)?
5. Has your appetite changed significantly lately?
6. Have you noticed any changes of sleep duration lately?
7. Have you noticed any changes in your interest in other people or things lately?
8. Have you been spending more money recently?
9. Have you recently noticed any change in your libido?
10. Has your feeling of being healthy or sick recently changed?

[a] Telephone visits were performed every two weeks and basing on the outcome of the talk, intervention visits were scheduled.
