# Supplementary material for: Behavioral and Self-reported Data Collected From Smartphones for the Assessment of Depressive and Manic Symptoms in Patients With Bipolar Disorder: Prospective Observational Study
Source: J Med Internet Res. 2022 Jan 19;24(1):e28647. doi: 10.2196/28647 (PMC8811705; doi:10.2196/28647)
Supplement: Multimedia Appendix 2 [file jmir_v24i1e28647_app2.pdf]

Regression coefficients from mixed models with changed ground truth for the analyses (3 days before visit and the day of the visit) to describe relation between smartphone-based data[a] collected using the BDmon application and depressive and manic symptoms assessed with the HDRS and YMRS, respectively.

	**Depressive symptoms (HDRS)**				**Manic symptoms (YMRS)**					
**Parameter**	Regression coef.	*P*	Confidence interval (95%)		Regression coef.	*P*	Confidence interval (95%)		**Number of Observations (patient-days)**	**Number of groups (patients)**
			lower limit	upper limit			lower limit	upper limit		
Number of incoming answered calls	−0.099	.177	−0.245	0.045	−0.11	.202	−0.278	0.059	382	46
Duration of incoming calls [s]	0.001	.246	−0.001	0.002	0.001	.893	−0.001	0.002	317	44
Standard deviation of duration of incoming calls [s]	0.001	.403	−0.001	0.003	0.002	.183	−0.001	0.004	226	40
Number of outgoing calls	−0.041	.211	−0.106	0.023	−0.027	.482	−0.102	0.049	382	46
Fraction of outgoing calls	−1.039	.361	−3.267	1.187	0.433	.741	−2.127	3.019	348	46
Duration of outgoing calls [s]	0.001	.364	−0.001	0.002	0.001	.412	−0.001	0.003	348	46
Standard deviation of duration of outgoing calls [s]	0.001	.440	−0.001	0.003	**0.004**	**.004**	**0.001**	**0.006**	300	44
Number of missed calls	0.071	.416	−0.101	0.243	**0.252**	**.015**	**0.05**	**0.453**	382	46
Fraction of missed calls	**4.944**	**.008**	**1.338**	**8.585**	2.742	.259	−2.073	7.487	232	42
Number of sent text messages	0.024	.086	−0.003	0.052	**0.132**	**<.001**	**0.103**	**0.162**	382	46
Mean length of text messages [# of chr]	0.001	.835	−0.012	0.015	**0.046**	**<.001**	**0.024**	**0.068**	127	23
Self-assessment of sleep time	**−0.453**	**.018**	**−0.821**	**−0.082**	−0.253	.098	−0.555	0.043	103	31
Self-assessment of mood	**−1.614**	**<.001**	**−2.135**	**−1.09**	**0.43**	**.028**	**0.052**	**0.817**	106	32

[a] In total, 382 patients-days of data collected from smartphones of 46 patients were relevant for this analysis
