# Supplementary material for: Behavioral and Self-reported Data Collected From Smartphones for the Assessment of Depressive and Manic Symptoms in Patients With Bipolar Disorder: Prospective Observational Study
Source: J Med Internet Res. 2022 Jan 19;24(1):e28647. doi: 10.2196/28647 (PMC8811705; doi:10.2196/28647)
Supplement: Multimedia Appendix 3 [file jmir_v24i1e28647_app3.pdf]

Regression coefficients from mixed models to describe relation between smartphone-based data collected using the BDmon application and depressive and manic symptoms assessed with the HDRS and YMRS, respectively with changed cut-off points on the HDRS and YMRS scales[a].

**Parameter**	**A. Euthymia vs Depression**						**B. Euthymia vs Mania**					
	Regression coeff.	*P*	Confidence intervals lower limit	Confidence intervals upper limit	**Number of Observations**	**Number of groups (patients)**	Regression coeff.	*P*	Confidence intervals lower limit	Confidence intervals upper limit	**Number of Observations**	**Number of groups (patients)**
Number of incoming answered calls	**−0.156**	**<.001**	**−0.157**	**−0.155**	877	49	−0.041	.438	−0.146	0.063	761	47
Duration of incoming calls [s]	0	.279	0	0.001	735	48	**0.001**	**.046**	**0**	**0.003**	647	45
Standard deviation of duration of incoming calls [s]	0	.862	−0.001	0.001	539	46	0.001	.217	0	0.002	488	42
Number of outgoing calls	**−0.073**	**.010**	**−0.129**	**−0.017**	877	49	0.009	.472	−0.015	0.033	761	47
Fraction of outgoing calls	−0.298	.701	−1.821	1.224	788	49	1.805	.124	−0.498	4.109	696	46
Duration of outgoing calls [s]	**0.002**	**.024**	**0**	**0.003**	788	49	0.001	.375	−0.001	0.002	696	46
Standard deviation of duration of outgoing calls [s]	0.001	.154	0	0.002	666	46	0.002	.059	0	0.003	606	44
Number of missed calls	**−0.013**	**<.001**	**−0.014**	**−0.011**	877	49	0.096	.153	−0.036	0.227	761	47
Fraction of missed calls	**2.547**	**.022**	**0.362**	**4.731**	508	48	**4.289**	**.010**	**1.022**	**7.557**	448	46
Number of sent text messages	**0.057**	**<.001**	**0.055**	**0.058**	877	49	**0.033**	**.005**	**0.01**	**0.056**	761	47
Mean length of text messages [# of chr]	0.008	.301	−0.007	0.022	289	32	**0.028**	**.011**	**0.006**	**0.049**	247	32
Self-assessment of sleep time	−0.035	.692	−0.206	0.137	243	36	−0.425	.338	−1.296	0.445	206	37
Self-assessment of mood	**−0.778**	**<.001**	**−1.152**	**−0.404**	247	39	1.83	.154	−0.687	4.348	206	38

[a] Euthymic state was define as a Hamilton Depression Rating Scale 17-items (HDRS-17) score < 13 and a Young Mania Rating Scale (YMRS) score < 13. A depressive state was define as a HDRS-17 score ≥ 13 and an YMRS score < 13. Manic state was defined as an YMRS score ≥ 13. Mixed state with higher cut offs was excluded from this analysis due to the relatively small sample size. In total, only 19 patient-days of the mixed state were reported for 2 patients.
