# Supplementary figures and images for: Behavioral and Self-reported Data Collected From Smartphones for the Assessment of Depressive and Manic Symptoms in Patients With Bipolar Disorder: Prospective Observational Study
Source: J Med Internet Res. 2022 Jan 19;24(1):e28647. doi: 10.2196/28647 (PMC8811705; doi:10.2196/28647)

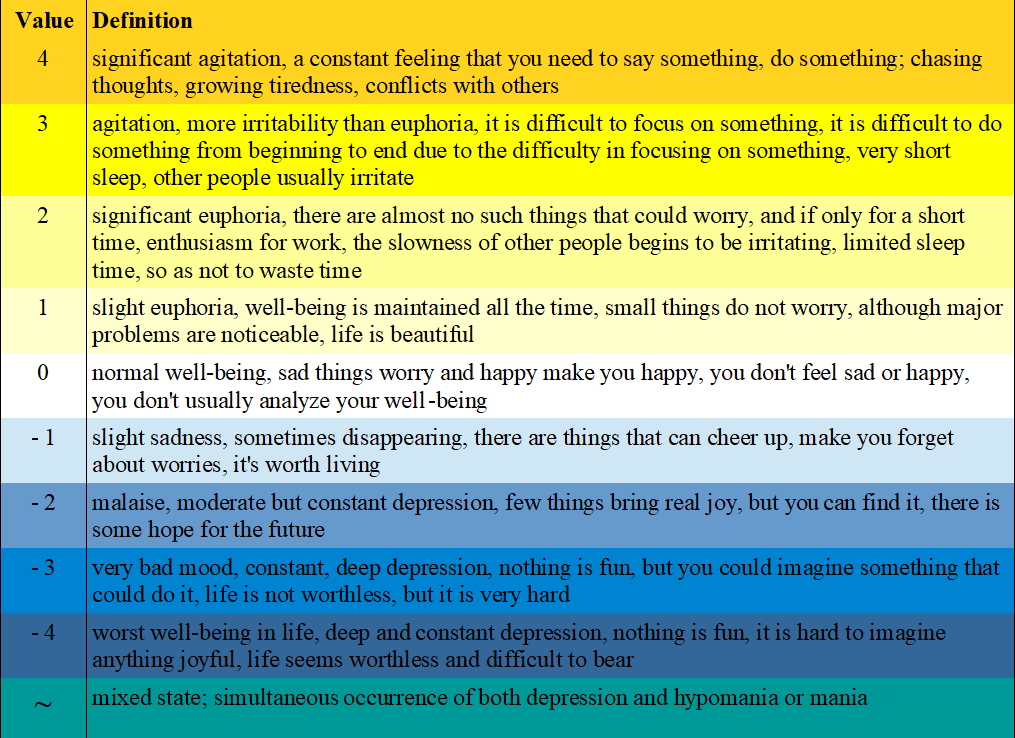

Supplement: Multimedia Appendix 4 [file jmir_v24i1e28647_app4.png]

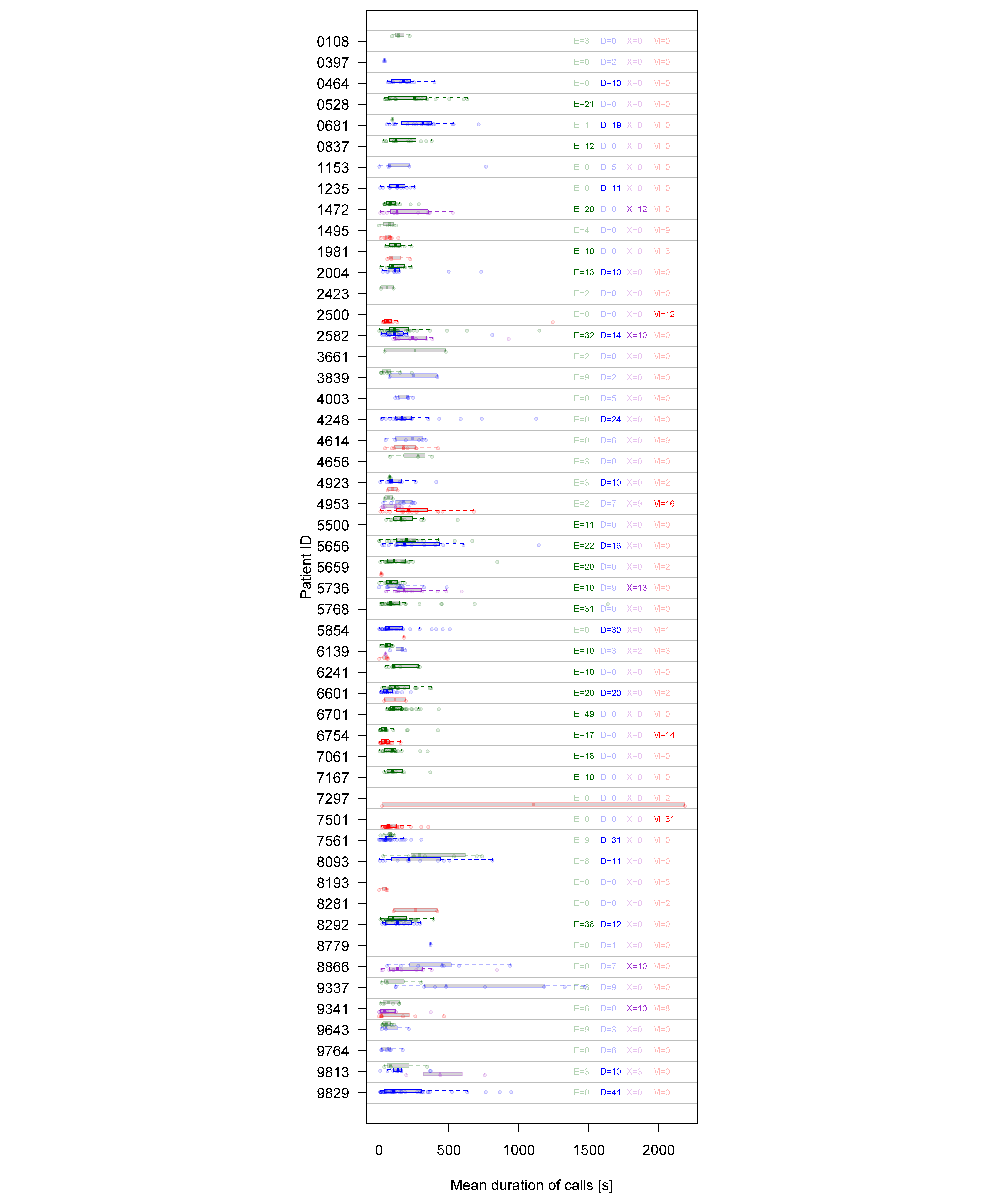

Supplement: Multimedia Appendix 8 [file jmir_v24i1e28647_app8.png]

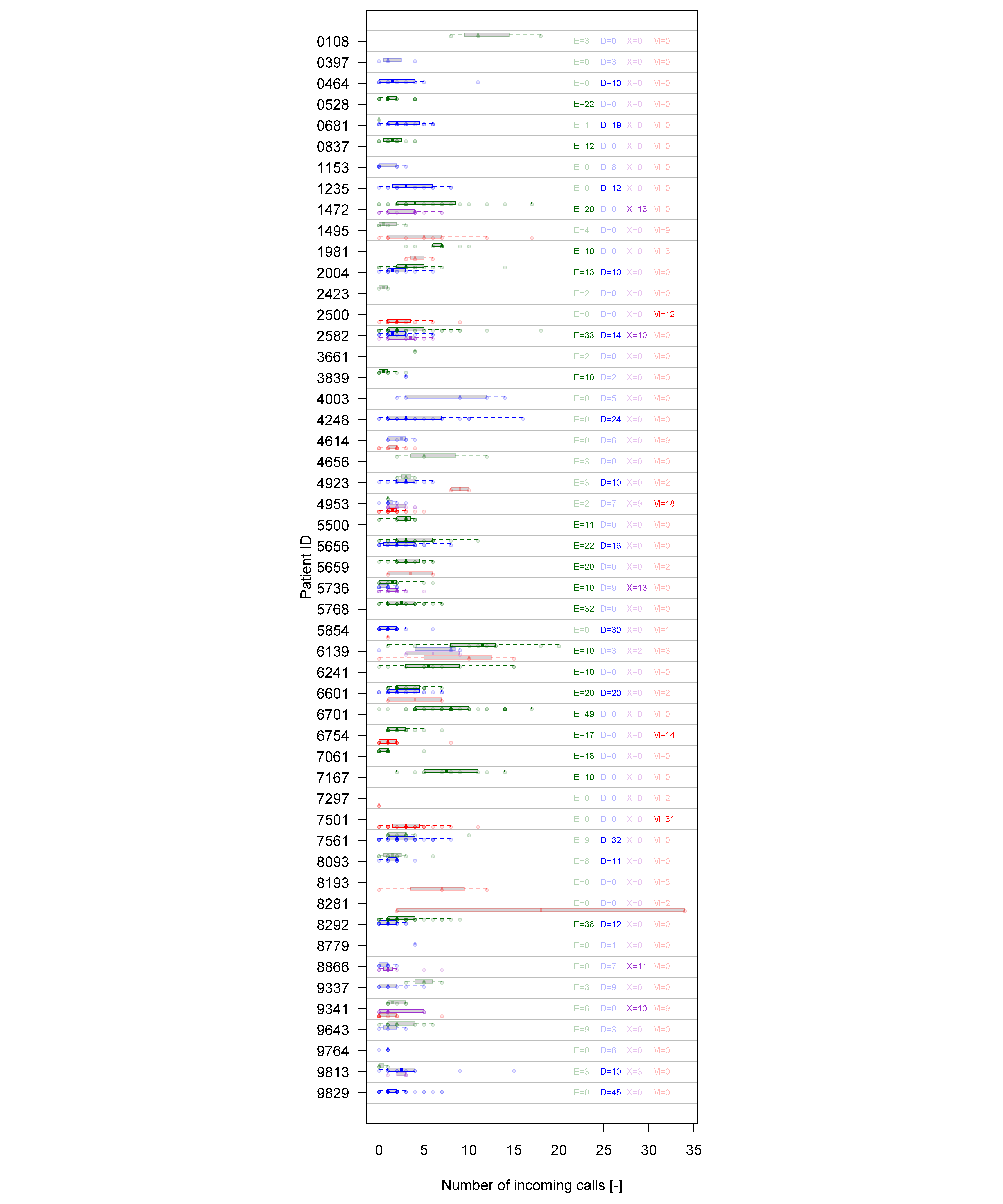

Supplement: Multimedia Appendix 9 [file jmir_v24i1e28647_app9.png]

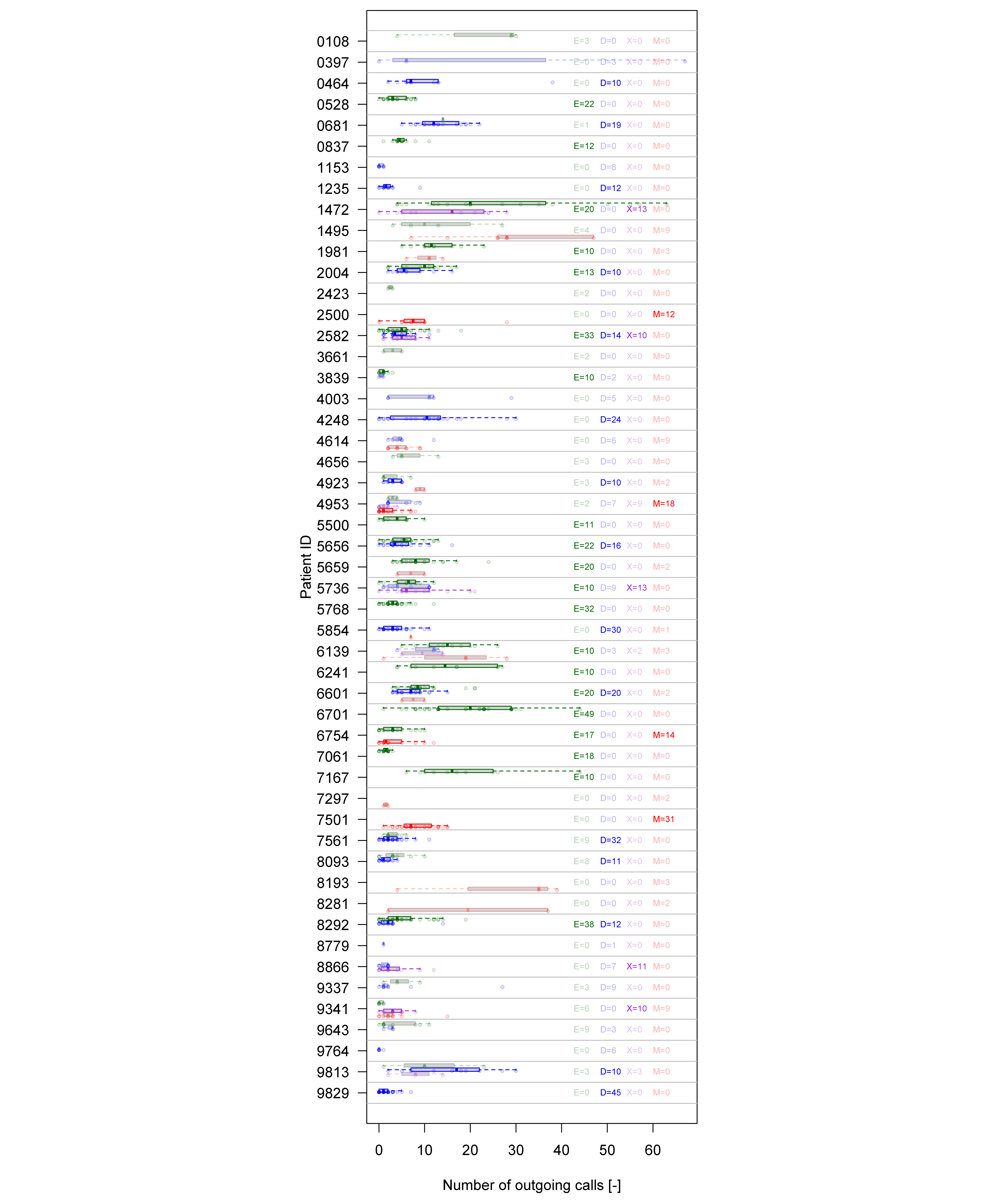

Supplement: Multimedia Appendix 10 [file jmir_v24i1e28647_app10.png]

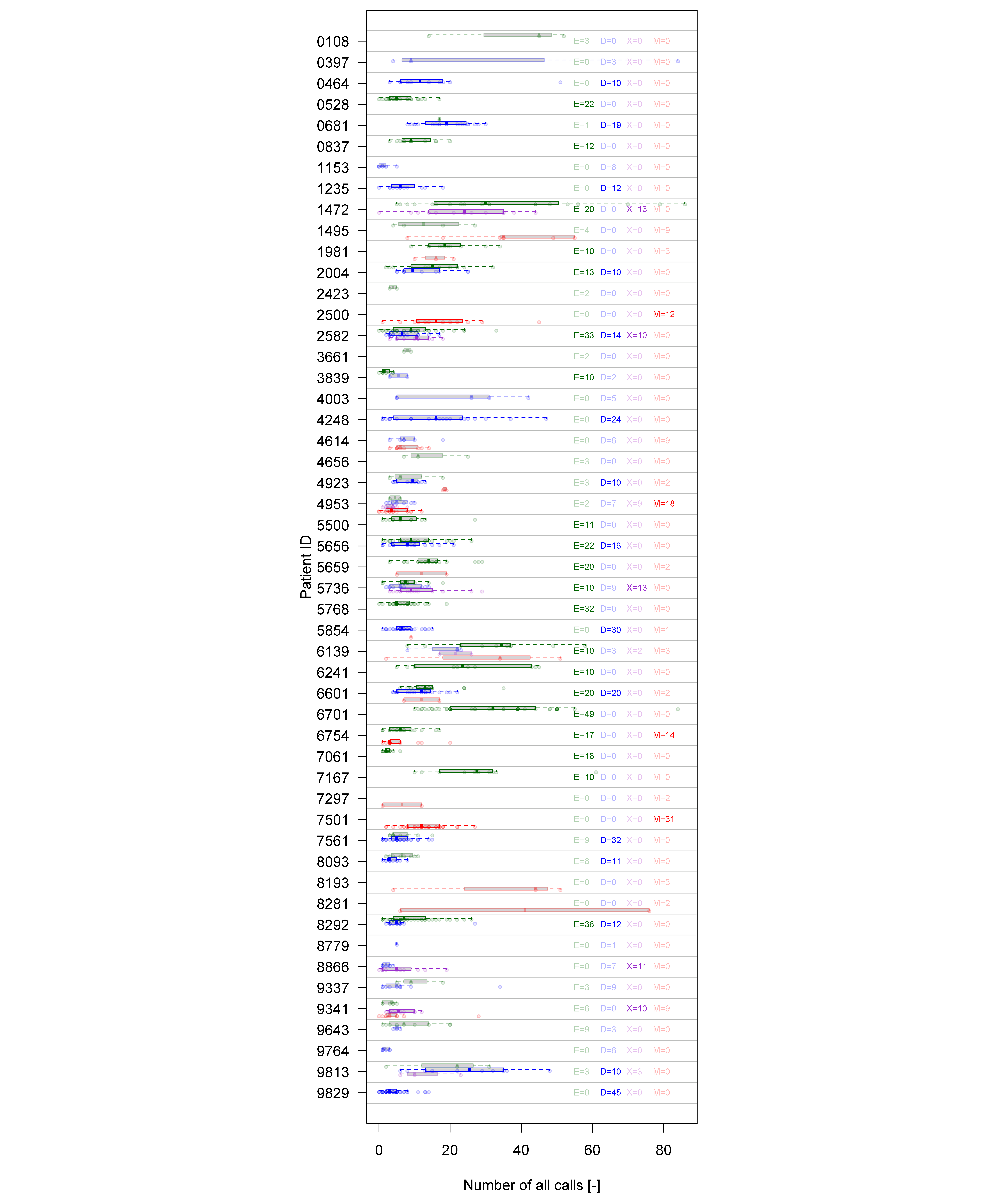

Supplement: Multimedia Appendix 11 [file jmir_v24i1e28647_app11.png]

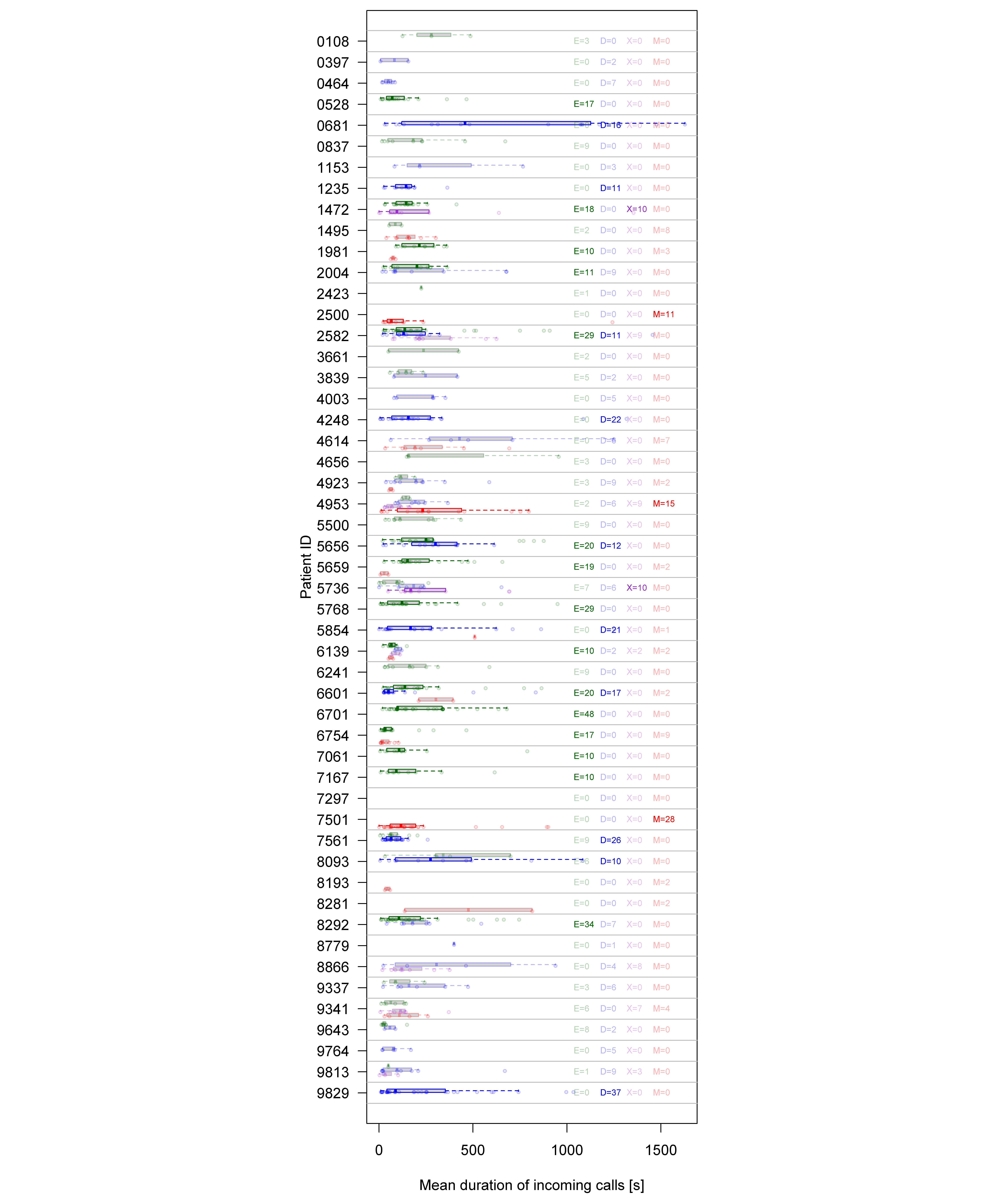

Supplement: Multimedia Appendix 12 [file jmir_v24i1e28647_app12.png]

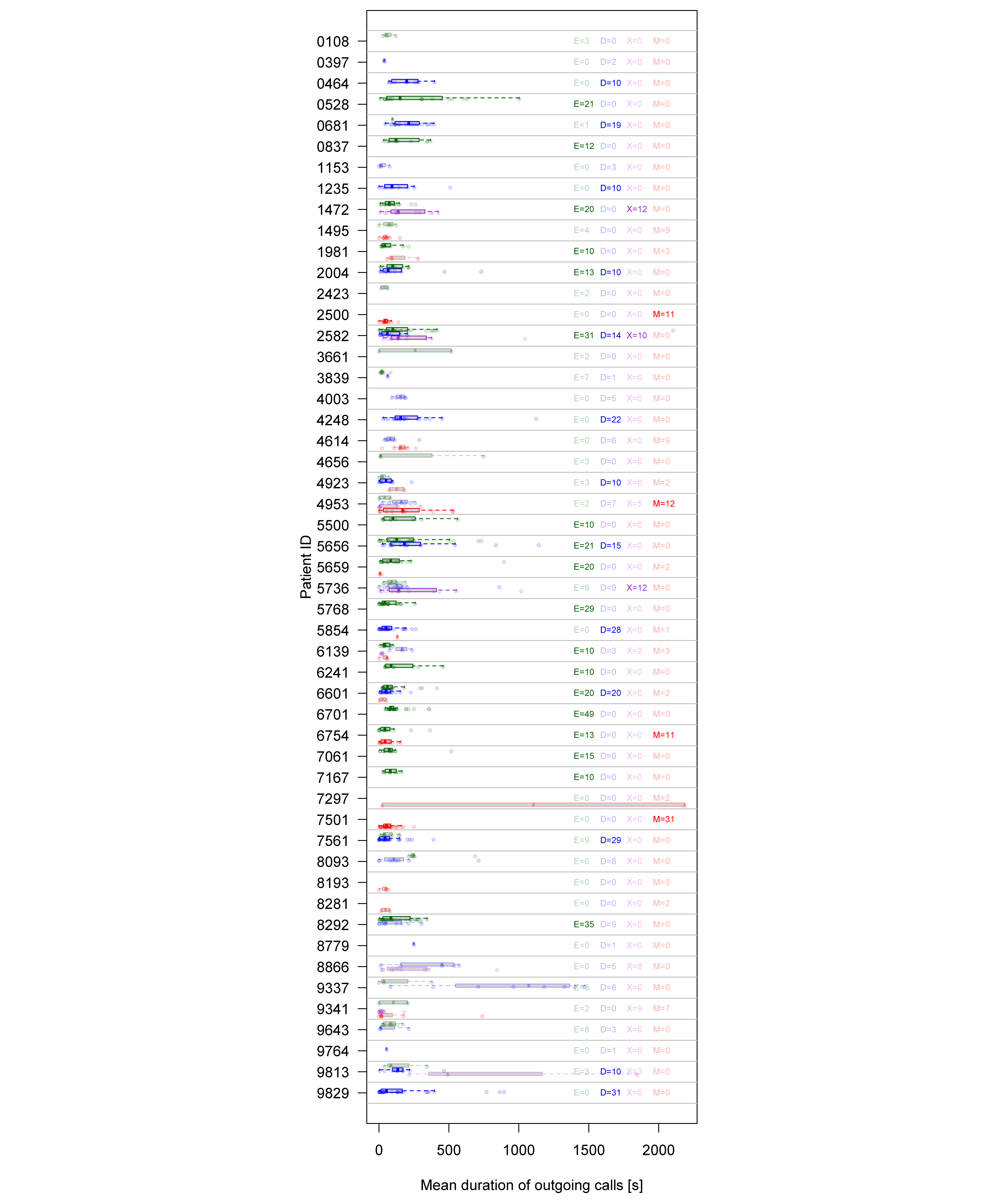

Supplement: Multimedia Appendix 13 [file jmir_v24i1e28647_app13.png]

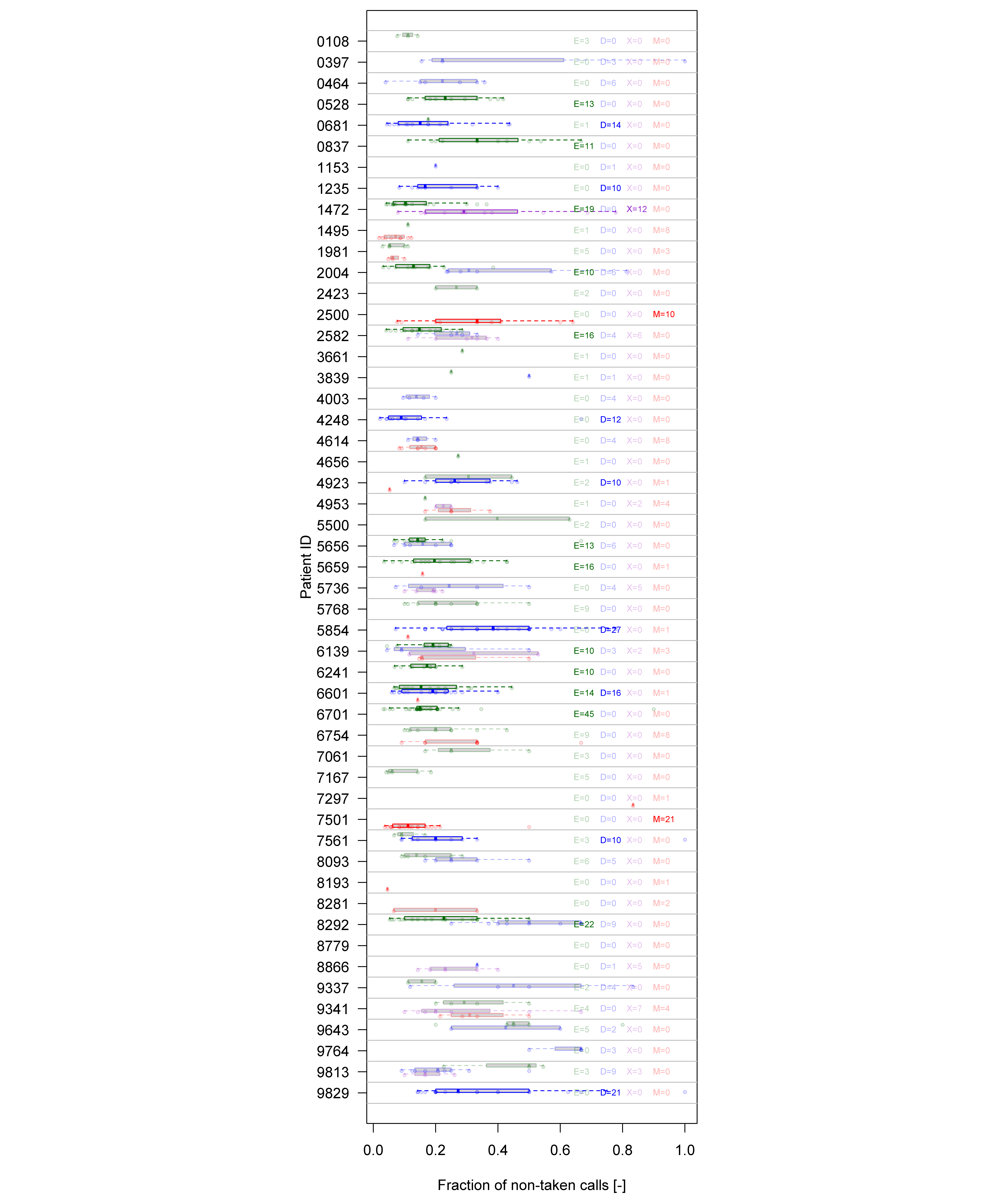

Supplement: Multimedia Appendix 14 [file jmir_v24i1e28647_app14.png]

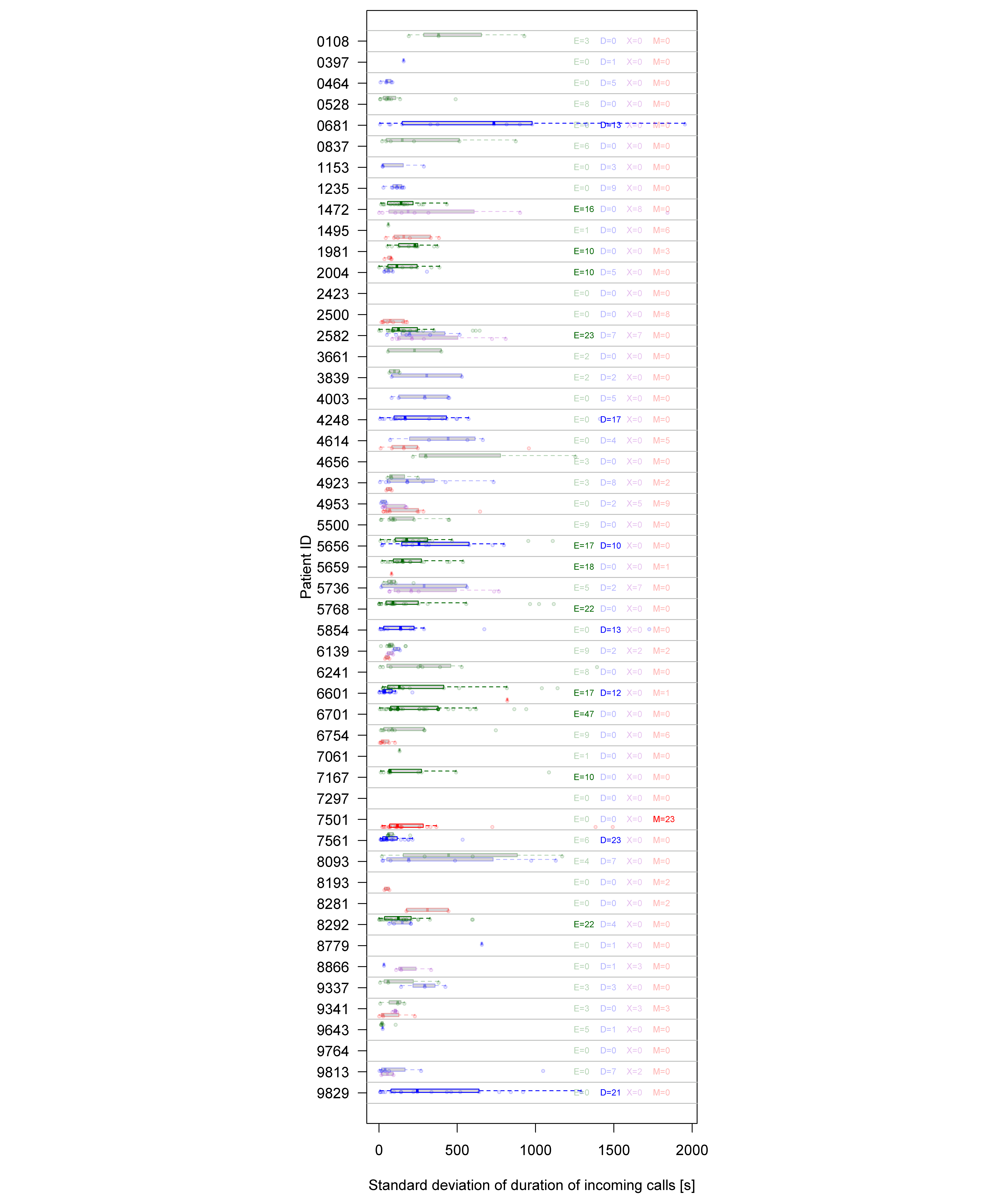

Supplement: Multimedia Appendix 15 [file jmir_v24i1e28647_app15.png]

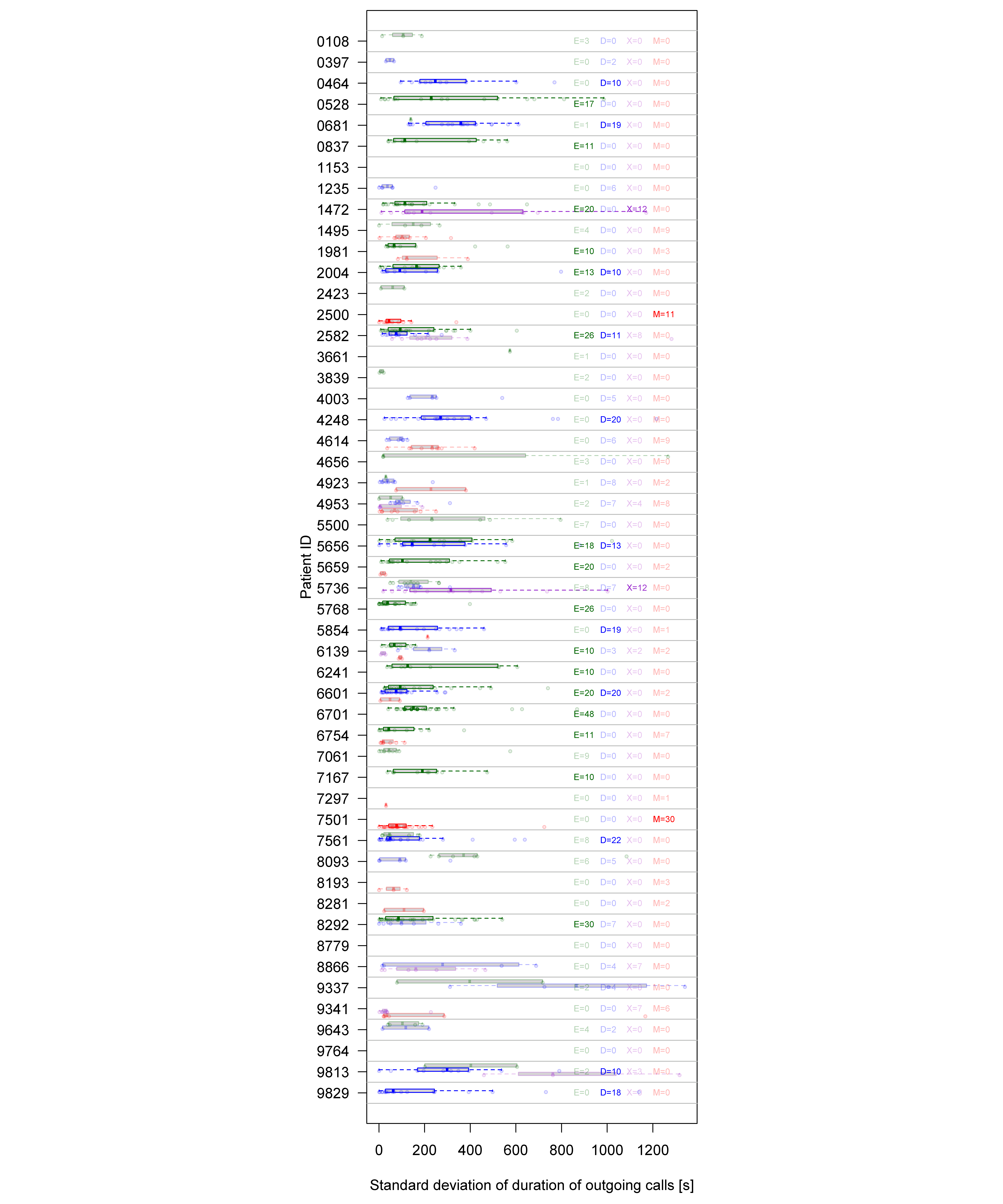

Supplement: Multimedia Appendix 16 [file jmir_v24i1e28647_app16.png]

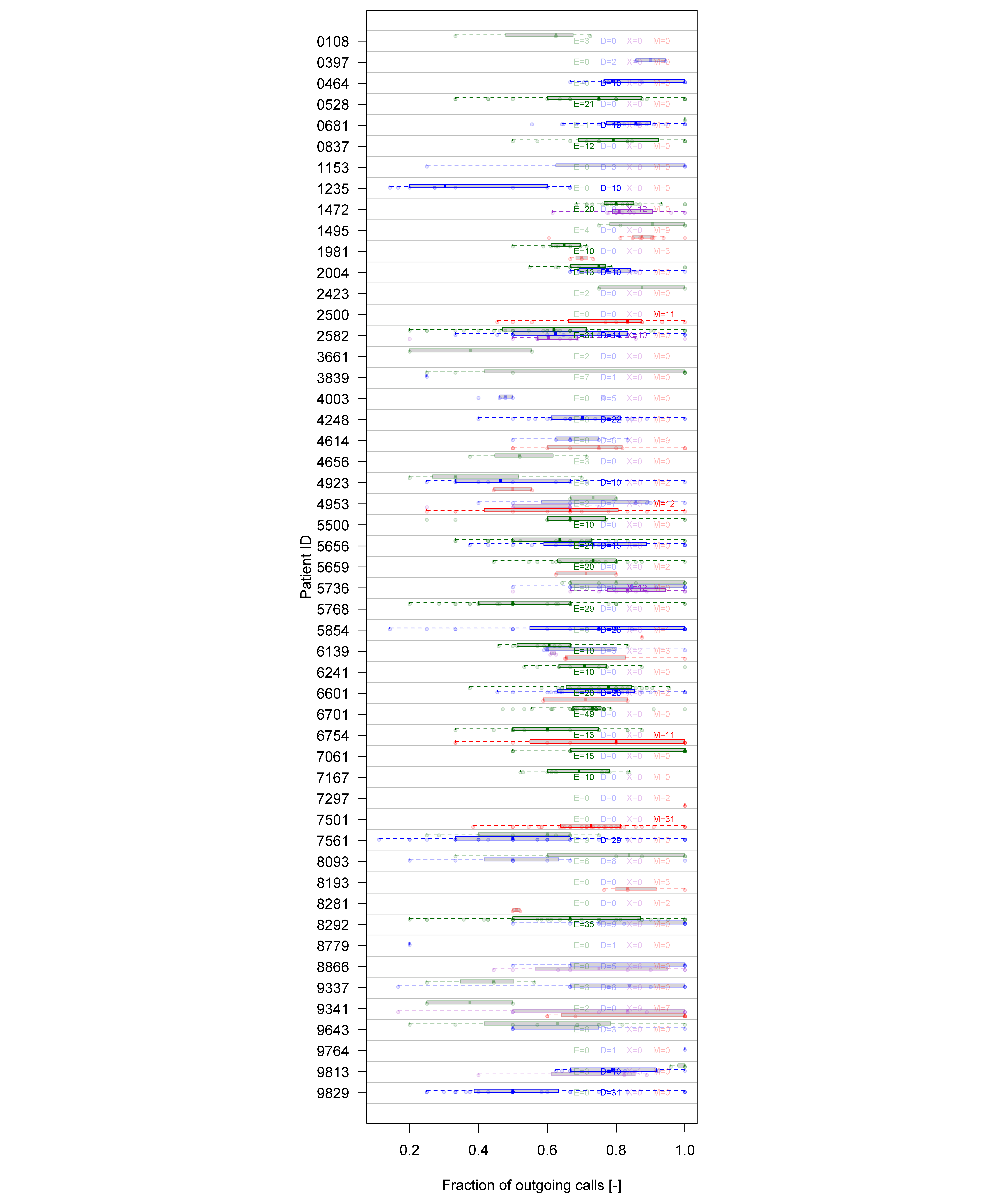

Supplement: Multimedia Appendix 17 [file jmir_v24i1e28647_app17.png]

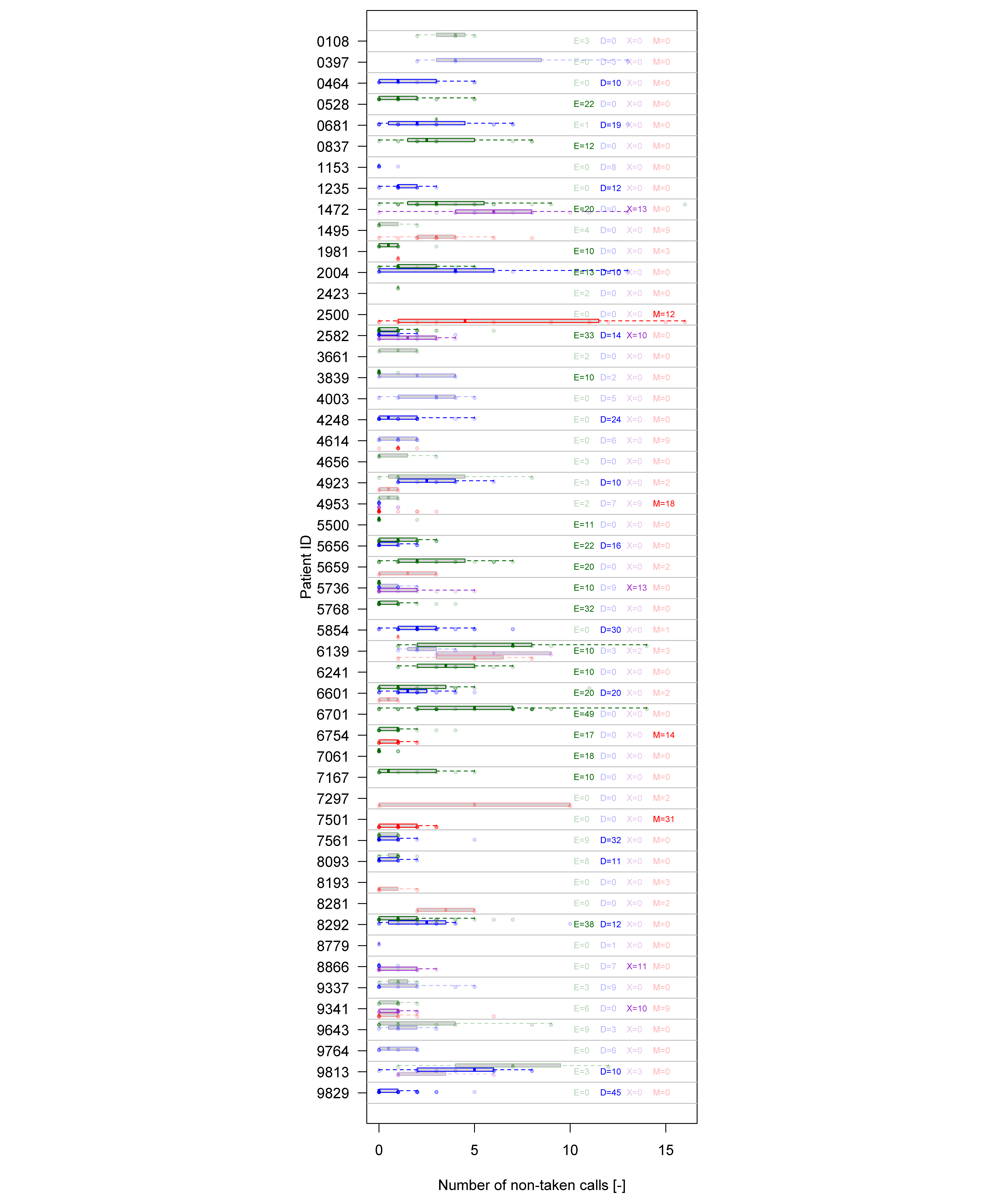

Supplement: Multimedia Appendix 18 [file jmir_v24i1e28647_app18.png]

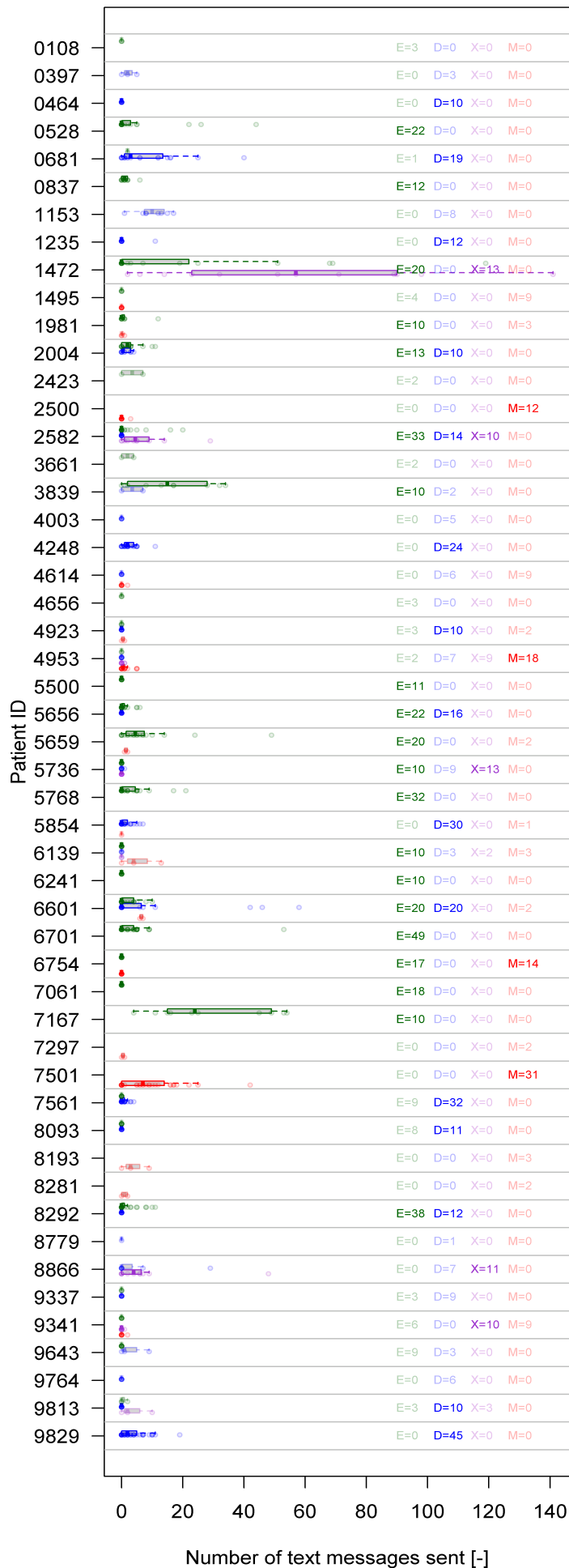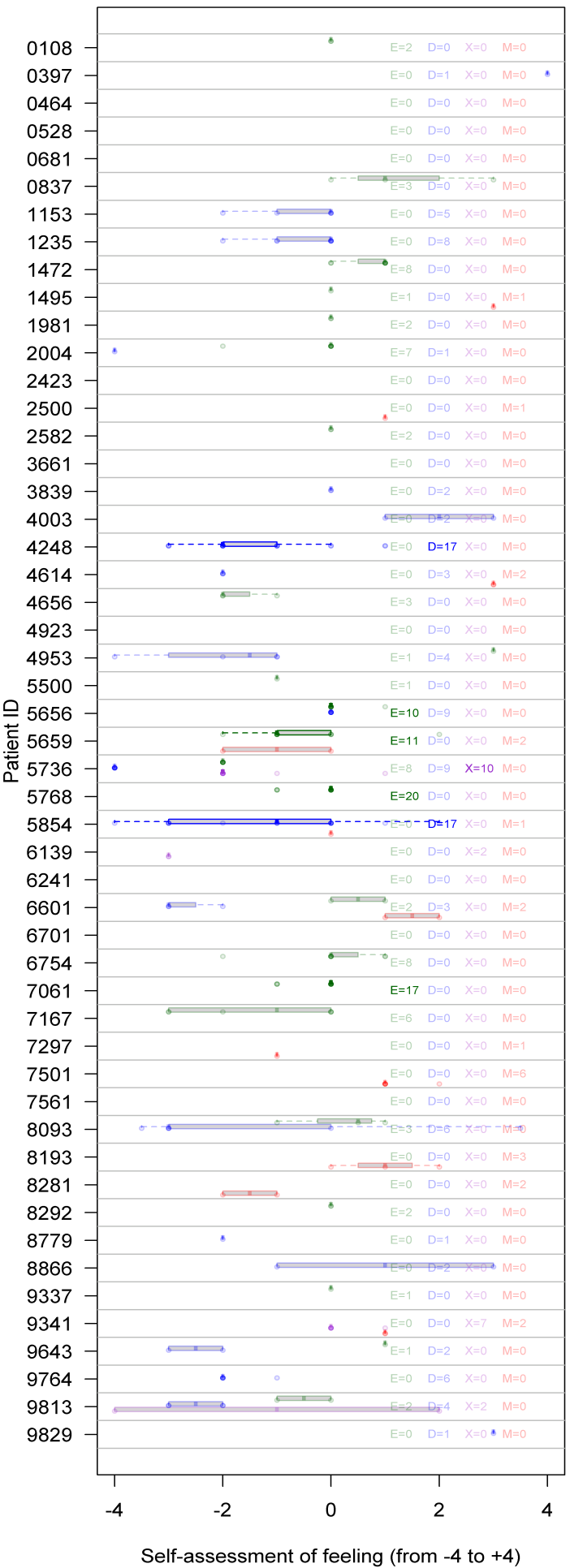

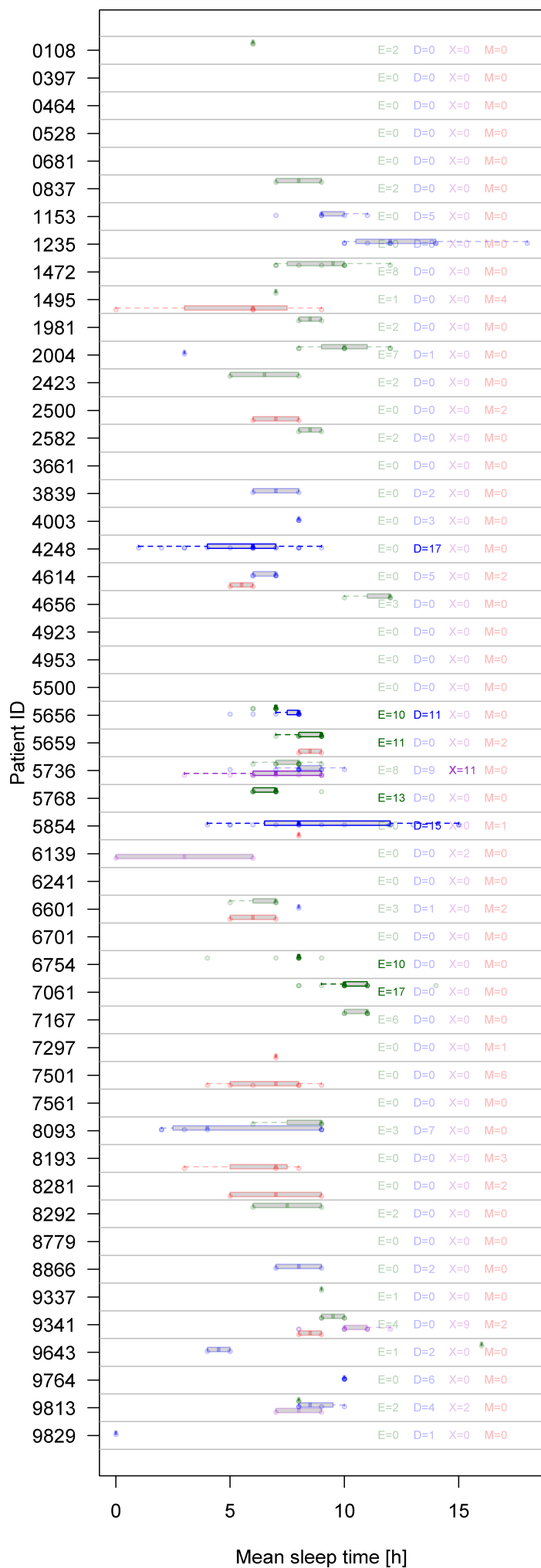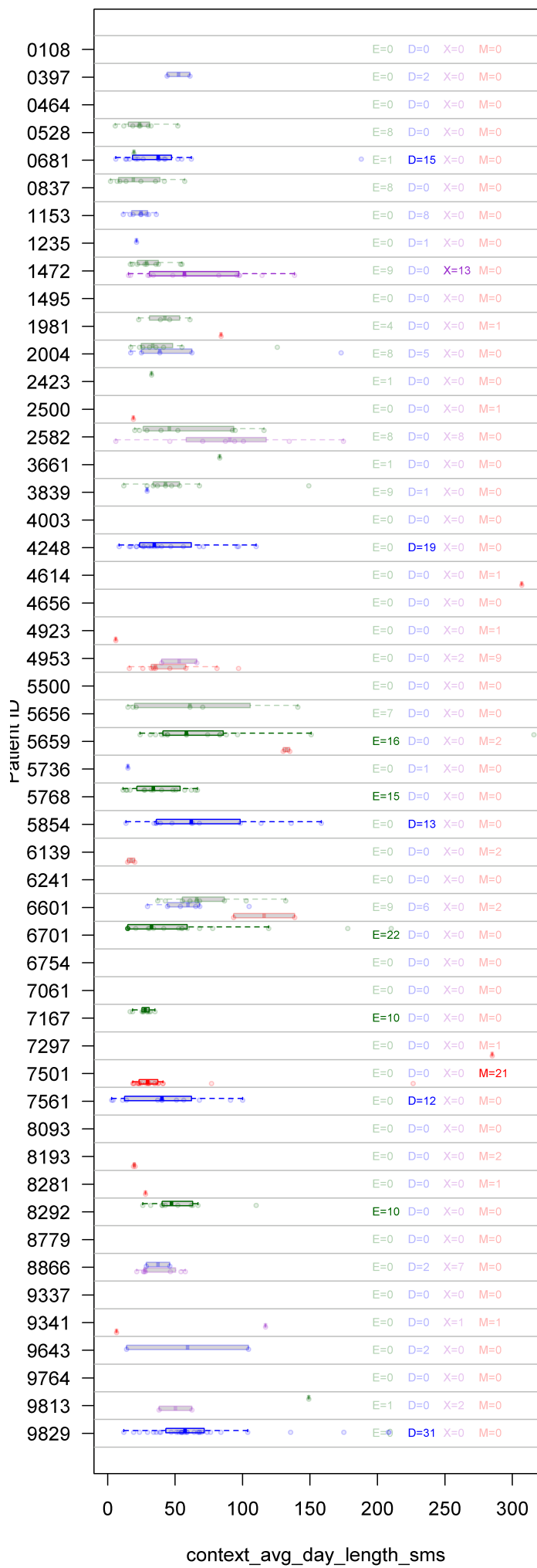

Supplement: Multimedia Appendix 19 [file jmir_v24i1e28647_app19.pdf]
