# Supplementary material for: Behavioral and Self-reported Data Collected From Smartphones for the Assessment of Depressive and Manic Symptoms in Patients With Bipolar Disorder: Prospective Observational Study
Source: J Med Internet Res. 2022 Jan 19;24(1):e28647. doi: 10.2196/28647 (PMC8811705; doi:10.2196/28647)
Supplement: Multimedia Appendix 7 [file jmir_v24i1e28647_app7.pdf]

		Euthymia			Depression			Mania			Mixed state		
	**Variable**	**Patient-days**	**Mean**	**SD**	**Patient-days**	**Mean**	**SD**	**Patient-days**	**Mean**	**SD**	**Patient-days**	**Mean**	**SD**
Phone calls	Number of incoming answered calls	445	3.9	3.8	344	2.4	2.6	122	3.2	4.3	71	2.4	2.1
	Duration of incoming calls [s]	390	188.0	225.0	274	252.3	373.8	98	191.3	237.5	58	194.4	232.2
	Standard deviation of duration of incoming calls [s]	302	212.5	248.8	188	282.1	421.6	73	189.0	280.9	37	262.3	361.1
	Number of outgoing calls	445	8.5	9.2	344	5.2	6.8	122	10.4	21.4	71	6.6	6.9
	Fraction of outgoing calls	416	0.7	0.2	299	0.7	0.2	110	0.7	0.2	61	0.7	0.2
	Duration of outgoing calls [s]	416	133.8	219.7	299	165.4	229.5	110	108.6	229.2	61	218.5	312.3
	Standard deviation of duration of outgoing calls [s]	365	189.8	241.8	238	214.8	250.1	98	119.9	155.8	55	290.8	331.7
	Number of missed calls	445	2.0	2.9	344	1.5	2.1	122	1.8	2.9	71	2.2	3.0
	Fraction of missed calls	268	0.2	0.1	195	0.3	0.2	77	0.2	0.2	42	0.3	0.2
Short messages	Number of sent text messages	445	3.5	10.7	344	2.2	6.2	122	2.9	6.2	71	15.0	37.0
	Mean length of text messages [# of chr]	147	51.4	49.0	118	53.6	41.5	45	57.4	67.4	33	65.3	41.0
Self-assessment	Self-assessment of sleep time	120	8.5	1.9	99	7.8	3.1	27	6.6	2.1	24	7.9	2.8
	Self-assessment of mood	121	−0.2	1.0	103	−1.3	1.8	23	0.8	1.4	21	−1.1	1.5

[a] The state of euthymia (E) was defined as HDRS < 8, YMRS < 6, depression (D): HDRS ≥ 8 and YMRS < 6, hypomania/mania (M): HDRS < 8, YMRS ≥ 6, mixed state (X): HDRS ≥ 8, YMRS ≥ 6
