# Supplementary material for: Behavioral and Self-reported Data Collected From Smartphones for the Assessment of Depressive and Manic Symptoms in Patients With Bipolar Disorder: Prospective Observational Study
Source: J Med Internet Res. 2022 Jan 19;24(1):e28647. doi: 10.2196/28647 (PMC8811705; doi:10.2196/28647)
Supplement: Multimedia Appendix 20 [file jmir_v24i1e28647_app20.pdf]

Regression coefficients from mixed regression models regarding smartphone-based data and affective states (euthymia vs depressive/mixed/manic states) in BD patients assessed with the HDRS and YMRS for a longer period before the psychiatric assessment (14 days) [a].

**Daily variable**	**Euthymia vs Depression/Mania/Mixed state**					
	Regression coeff.	*P*	Confidence intervals		**No. of observations**	**No.of groups (patients)**
			Lower limit	Upper limit		
**Number of incoming answered calls**	**-0.058**	**.03**	**-0.111**	**-0.005**	**1511**	**52**
Duration of incoming calls [seconds/call]	0.000	.21	0	0.001	1275	52
Standard deviation of duration of incoming calls [s]	0.001	.11	0	0.001	928	49
Number of outgoing calls	0.007	.41	-0.009	0.022	1511	52
**Fraction of outgoing calls**	**1.012**	**.01**	**0.218**	**1.806**	**1361**	**52**
Duration of outgoing calls [s]	0.001	.08	0	0.001	1361	52
Standard deviation of duration of outgoing calls [s]	0.000	.375	0	0.001	1154	51
Number of missed calls	0.043	.22	-0.026	0.112	1511	52
**Fraction of missed calls**	**2.412**	**<.001**	**1.083**	**3.741**	**875**	**51**
**Number of sent text messages**	**0.021**	**.01**	**0.005**	**0.037**	**1511**	**52**
**Mean length of text messages [no. of characters]**	**0.010**	**.04**	**0**	**0.02**	**343**	**38**
**Self-assessment of sleep time [hours]**	-0.081	.30	-0.236	0.073	409	40
**Self-assessment of mood**	**-0.617**	**<.001**	**-0.945**	**-0.288**	**408**	**43**

[a] Euthymic state was define as a Hamilton Depression Rating Scale 17-items (HDRS-17) score < 13 and a Young Mania Rating Scale (YMRS) score < 13. A depressive state was define as a HDRS-17 score ≥ 13 and an YMRS score < 13. Manic state was defined as an YMRS score ≥ 13. Mixed state with higher cut offs was excluded from this analysis due to the relatively small sample size. In total, only 19 patient-days of the mixed state were reported for 2 patients.
